# Two Compact Cas9 Ortholog-Based Cytosine Base Editors Expand the DNA Targeting Scope and Applications *In Vitro* and *In Vivo*

**DOI:** 10.3389/fcell.2022.809922

**Published:** 2022-03-01

**Authors:** Susu Wu, Liping Li, Min Li, Shiyu Sun, Yuting Zhao, Xiaowen Xue, Feiyu Chen, Jingli Zhong, Junfan Guo, Qianhui Qu, Xiongjun Wang, Zhen Liu, Yunbo Qiao

**Affiliations:** ^1^ Precise Genome Engineering Center, School of Life Sciences, Guangzhou University, Guangzhou, China; ^2^ Institute of Neuroscience, CAS Center for Excellence in Brain Science and Intelligence Technology, CAS Key Laboratory of Primate Neurobiology, State Key Laboratory of Neuroscience, Chinese Academy of Sciences, Shanghai, China; ^3^ College of Life Sciences, University of Chinese Academy of Sciences, Beijing, China; ^4^ School of Life Science and Technology, ShanghaiTech University, Shanghai, China; ^5^ Shanghai Stomatological Hospital, Institutes of Biomedical Science, Department of Systems Biology for Medicine, Fudan University, Shanghai, China

**Keywords:** CRISPR/Cas9, ortholog, cytosine base editor, AR, disease modeling, SgoCas9, Sth1aCas9

## Abstract

CRISPR/Cas9-based base editing tools enable precise genomic installation and hold great promise for gene therapy, whereas the big size of Cas9 nucleases and its reliability on specific protospacer adjacent motif (PAM) sequences as well as target site preferences restrict the extensive applications of base editing tools. Here, we generate two cytosine base editors (CBEs) by fusing cytidine deaminases with two compact codon-optimized Cas9 orthologs from *Streptococcus_gordonii_str._Challis_substr._CH1* (ancSgo-BE4) and *Streptococcus_thermophilus_LMG_18311* (ancSth1a-BE4), which are much smaller than *Streptococcus pyogenes* (SpCas9) and recognize NNAAAG and NHGYRAA PAM sequences, respectively. Both CBEs display high activity, high fidelity, a different editing window, and low by-products for cytosine base editing with minimal DNA and RNA off-targeting activities in mammalian cells. Moreover, both editors show comparable or higher editing efficiencies than two engineered SpCas9 variant (SpCas9-NG and SpRY)-based CBEs in our tested target sites, which perfectly match the PAM sequences for ancSgo-BE4 or ancSth1a-BE4. In addition, we successfully generate two mouse models harboring clinically relevant mutations at the *Ar* gene *via* ancSgo-BE4 and ancSth1a-BE4, which display androgen insensitivity syndrome and/or developmental lethality in founder mice. Thus, the two novel CBEs broaden the base editing tool kits with expanded targeting scope and window for efficient gene modification and applications, respectively.

## Introduction

Base editor (BE) systems, including cytosine base editors (CBEs) and adenine base editors (ABEs), can induce C-to-T and A-to-G substitutions efficiently in cultured cells, animals, and plants (Komor et al., 2016; Gaudelli et al., 2017; Kim et al., 2017; Zong et al., 2017). Applications of base editing tools largely facilitate disease modeling, functional analyses, disease therapy (Liu et al., 2018; Yang et al., 2019; Despres et al., 2020; Jeong et al., 2020; Yeh et al., 2020; Cuella-Martin et al., 2021; Koblan et al., 2021), *etc*. CBEs and ABEs, which are originally designed by fusing DNA deaminases with a Cas9 nickase (Cas9n), can efficiently induce target base conversions without double-strand DNA breaks (Komor et al., 2016; Gaudelli et al., 2017). To resolve the limitations of BE applications regarding editing efficiency, targeting scope, protospacer adjacent motif (PAM) sequence specificity, off-targeting activities, and product purity, a large set of engineered BEs with optimized features have been reported by fusing differential types of engineered Cas9 variants with optimized deaminases or fusing their orthologs from different organisms (Rees and Liu, 2018; Cheng et al., 2019; Anzalone et al., 2020; Porto et al., 2020). For instance, classical CBEs developed from *Streptococcus pyogenes* (SpCas9) prefer the target bases at position 4–8 within the protospacer with NGG PAM (Jinek et al., 2012; Jiang et al., 2013; Kleinstiver et al., 2015), and substitution of SpCas9n with engineered variants or Cas9 orthologs can potentially alter the targeting scope, PAM preference, molecular size, and editing features (Esvelt et al., 2013; Chatterjee et al., 2018; Li et al., 2018; Rousseau et al., 2018; Huang S. et al., 2019; Doman et al., 2020; Hu et al., 2020; Walton et al., 2020).

Although the applications of engineered PAM-less SpRY Cas9 potentially and extremely expand the genome editing scope without PAM restriction (Ren et al., 2021; Xu et al., 2021), the editing efficiency of different BEs at different genomic loci is commonly affected by the diversity of the microenvironment and epigenetic states in different cell types or tissues, with various editing levels (Liu et al., 2019; Anzalone et al., 2020), which is commonly referred as “site selection.” Therefore, developing novel BEs with different PAM recognition, targeting window, and smaller size will be favorable for genome editing and gene therapy. Here, we develop two novel CBEs by fusing ancestral reconstructed APOBEC1 (ancAPOBEC1) (Koblan et al., 2018) with two Cas9 orthologs, SgoCas9 from *Streptococcus_gordonii_str._Challis_substr._CH1* and Sth1aCas9 from *Streptococcus_thermophilus_LMG_18311*, respectively (Gasiunas et al., 2020), which are named as ancSgo-BE4 and ancSth1a-BE4, respectively. Both CBEs display smaller size, high activity, high fidelity, specific PAM, and editing window different from reported CBEs (Jeong et al., 2020), low byproducts for cytosine base editing, as well as partial superiority over SpCas9-NG- and SpRY-based CBEs (Huang S. et al., 2019; Ren et al., 2021). Using the two novel CBEs, we also successfully generate two mouse models harboring clinically relevant mutations and phenotypes. These two CBEs broaden the repertoire and choice of base editing toolbox and expand the potential applications of base editors.

## Results

### Identification of ancSgo-BE4 and ancSth1a-BE4 as Two Novel CBEs

**FIGURE 1:**
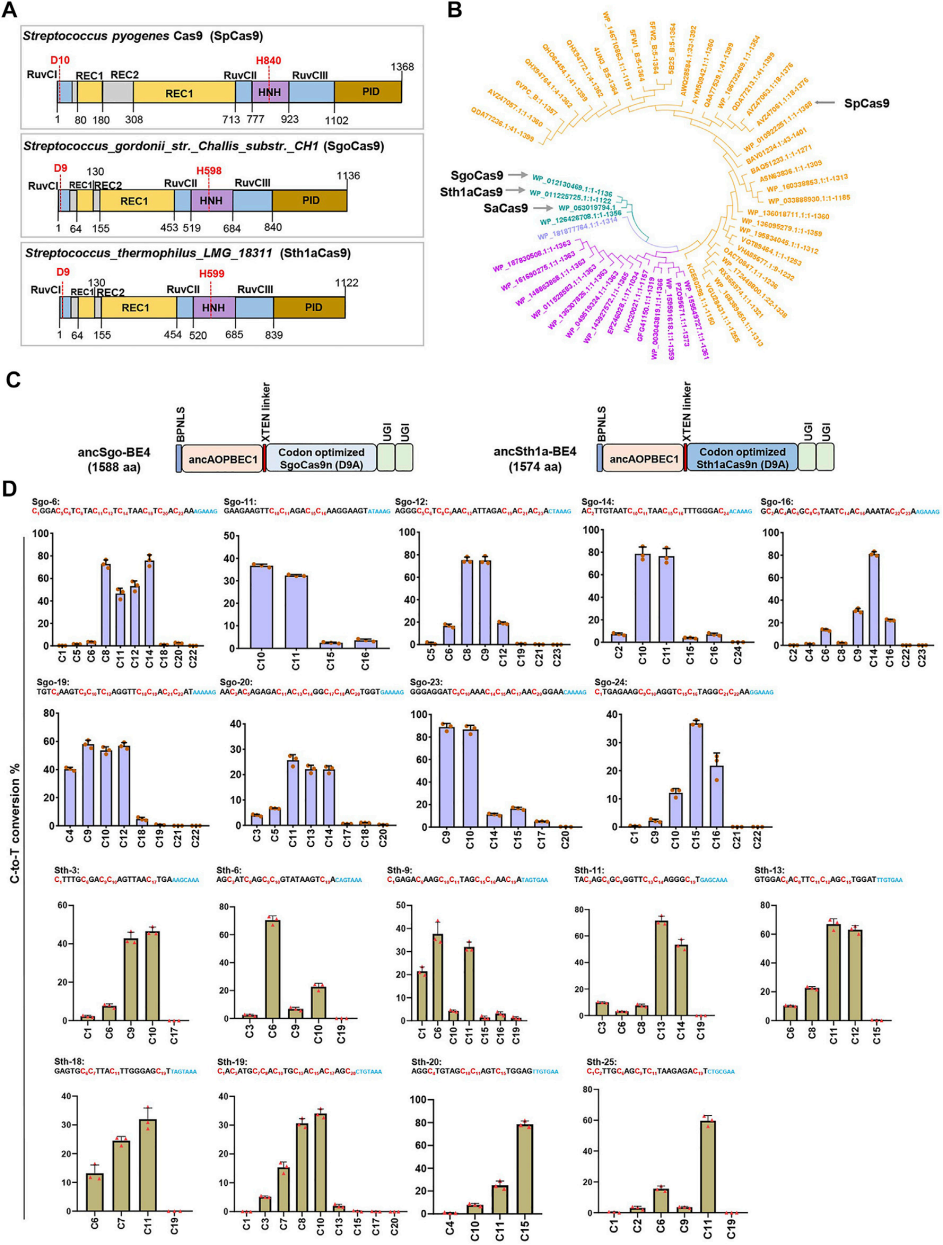
Construction of two novel CBEs. **(A)** Architectures of the SpCas9, SgoCas9, and Sth1aCas9. **(B)** Unrooted phylogenetic tree of selected Cas9 orthologs from different bacterial strains for activity screening. Four specified Cas9 orthologs (spCas9, saCas9, SgoCas9, and Sth1aCas9) are indicated. **(C)** Schematic diagram showing plasmid architectures of ancSgo-BE4 and ancSth1a-BE4. **(D)** The C-to-T conversion rates induced by ancSgo-BE4 and ancSth1a-BE4 at indicated target DNA sites in HEK293T cells were presented. Error bars represent standard error from three independent experiments. The sequences represent gRNA target and PAM sequences (blue), and the cytosines are ordered and highlighted in red.

Engineering of the bacterial CRISPR/Cas (clustered regularly interspersed short palindromic repeats/CRISPR-associated proteins) system as site-specific editors is an attractive strategy to expand the toolbox for genome editing. To expand the application of base editors with different PAM recognition, we referred to a Cas9 catalog with clarified cleavage characteristics in *in vitro* biochemical assays (Gasiunas et al., 2020). We selected Cas9 orthologs for BE engineering from 79 Cas9 proteins (Gasiunas et al., 2020) following the criteria: clear PAM sequences distinct with SpCas9, smaller than SpCas9, and high cleavage activity at 37°C. Finally, we designated two Cas9 orthologs, SgoCas9 (1,136 amino acids) and Sth1aCas9 (1,122 amino acids), which are much smaller than SpCas9 (1,368 amino acids) (Figure 1A). Protein sequence alignment revealed that SgoCas9 and Sth1aCas9 have 13.4 and 13.1% sequence identity with SpCas9, respectively (Supplementary Figure S1), and functional domains, including REC, RuvC, PID, and HNH domains, were designated according to sequence similarity (Supplementary Figure S1), with two conserved catalytic residues within RuvC and HNH nuclease domains (D9 and H598 for SgoCas9; D9 and H599 for Sth1aCas9) (Figure 1A).

Taking the protein sequence of SpCas9 as an input, we searched for Cas9 protein homologs with >80% homology and added *Staphylococcus aureus* Cas9 (SaCas9), SgoCas9, and Sth1aCas9 to construct a phylogenetic tree, demonstrating that SgoCas9 and Sth1aCas9 were quite close to SaCas9 (Figure 1B). We also compared the protein sequences of Sth1aCas9 with Sth1Cas9 (1,121 amino acids also named as St1Cas9) from *Streptococcus thermophilus* (Briner et al., 2014; Xu et al., 2015), showing that the two Cas9 proteins were highly similar, with only 34 different amino acids mainly distributed in PAM-interacting domains (PIDs) (Supplementary Figure S2A), which may lead to the difference of PAM recognition (Paez-Espino et al., 2015). Moreover, we also predicted the three-dimensional structure of SgoCas9 and Sth1aCas9, and compared them with the crystal structures of Sth1Cas9 (6M0V) (Zhang et al., 2020) and SpCas9 (4UN3) (Anders et al., 2014) (Supplementary Figures S3A–D). It demonstrated that the predicted structures of SgoCas9 and Sth1aCas9 were quite similar to that of Sth1Cas9; in particular, the structures of PIDs within these Cas9 proteins were remarkably different, which may reflect the difference of PAM recognition (Anders et al., 2014).

To explore the editing ability of the SgoCas9- and Sth1aCas9-based BE4 system (Koblan et al., 2018), we fused the nickase form (D9A) of eukaryotic codon-optimized SgoCas9 or Sth1aCas9 with ancAPOBEC1 to generate two CBEs, ancSgo-BE4 and ancSth1a-BE4 (Figure 1C). We also compared the scaffold sequences of guide RNAs (gRNAs) for SgoCas9 and Sth1aCas9 with those for SpCas9, showing 34 and 42% of conserved nucleotides, respectively (Supplementary Figure S2B). To test the editing efficiency of the two editors, we randomly chose some endogenous target sites (24-nt protospacer for SgoCas9 and 20-nt protospacer for Sth1aCas9) with predicted PAM sequences (“NNAAAG” for SgoCas9 and “NHGYRAA” for Sth1a) from *in vitro* biochemical assays (Supplementary Figure S2C) (Gasiunas et al., 2020). ancSgo-BE4 or ancSth1a-BE4 were co-transfected with their targeting gRNAs into HEK293T cells, and transfection-positive cells were collected for the detection of editing efficiency at 72 h. Among the tested sites with successful PCR amplification and Sanger sequencing, ancSgo-BE4 showed efficient editing at 18 genomic sites with frequencies of C-to-T conversion ranging from 4.6 to 90%, which were evaluated from Sanger sequence chromatograms using EditR (Brinkman et al., 2014), and the targeting efficiency was more than 30% for most target sites; ancSth1a-BE4 showed efficient editing at 9 detected target sites with 30–78.5% of the C-to-T conversion rate (Supplementary Figures S4A,B). To further validate the editing efficiencies and features of ancSgo-BE4 and ancSth1a-BE4, the PCR amplicons from 9 representative target sites with relatively higher editing efficiencies were subjected to targeted deep sequencing and analysis. Consistently, both ancSgo-BE4 and ancSth1a-BE4 displayed high C-to-T editing efficiency up to 85.1 and 81.8%, respectively (Figure 1D), which was comparable with the reported editing efficiency of BE4 (Koblan et al., 2018). Meanwhile, we amplified the wild-type HEK293T genome sequences of tested target sites and performed target deep sequencing, and the results showed that all tested loci did not show obvious editing efficiencies (Supplementary Figure S4C). These data suggest that our newly generated CBEs, ancSgo-BE4 and ancSth1a-BE4, can induce efficient cytosine base editing with NNAAAG and NHGYRAA PAM compatibilities, respectively, in endogenous human genomic loci.

### Characterization of ancSgo-BE4 and ancSth1a-BE4 as CBEs

**FIGURE 2:**
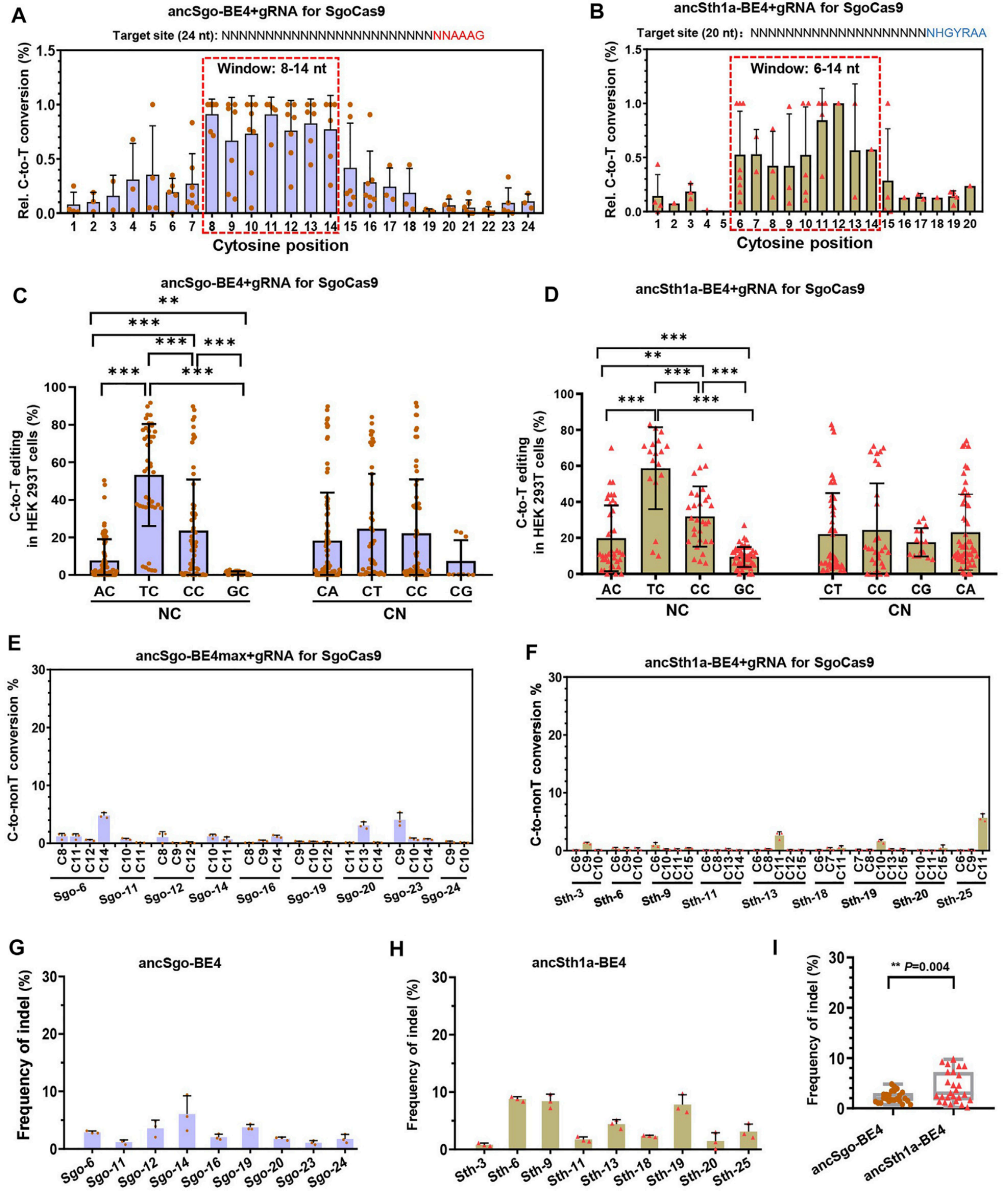
Characterization of editing features induced by ancSgo-BE4 and ancSth1a-BE4. **(A,B)** The C-to-T conversion rate for the cytosine with the highest efficiency for each site was normalized to “1”, and relative (Rel.) editing efficiencies for all cytosines at their position within the protospacer were presented. The most distal nucleotide relative to PAM was set as position “1.” The base editing windows for ancSgo-BE4 **(A)** and ancSth1a-BE4 **(B)** are highlighted by the dotted red box. **(C,D)** Comparison of base editing activity induced by ancSgo-BE4 **(C)** and ancSth1a-BE4 **(D)** in different neighboring contexts at the 5’ (NC) or 3’ (CN) ends of targeted cytosines (Student’s *t*-test). **p < 0.01, ***p < 0.001. **(E,F)** C-to-non-T (C-to-G and C-to-A) editing efficiency at 9 targeting sites induced by ancSgo-BE4 **(E)** and ancSth1a-BE4 **(F)** in HEK293T cells. Data were generated from targeted deep sequencing from three independent replicates. **(G,H)** Frequency of indels at 9 targeting sites induced by ancSgo-BE4 **(G)** and ancSth1a-BE4 **(H)** in HEK293T cells. **(I)** Comparison of frequencies of indels at 9 targeting sites in **G** and **H**. *P*-value was calculated from unpaired, two-tailed Student’s *t*-test. **p < 0.01.

Next, we characterized the editing features, preferences, and by-products of ancSgo-BE4 and ancSth1a-BE4. To clarify the editing window of the two CBEs, the editing efficiencies of all cytosines (C) within the protospacer were calculated, and the efficiency of the highest edited “C” for each target site was normalized to “1.” When the efficiencies of all cytosines were displayed together, we clearly observed that ancSgo-BE4 can efficiently induce base editing within a window ranging from positions 8 to 14 in the protospacer (setting the base distal to PAM as position 1) and that ancSth1a-BE4 can induce apparent cytosine base conversion within bases 6–14 of the 20-nt protospacer (Figures 2A,B), both of which were different from the editing window of BE4 (position 4–8 within the protospacer) (Koblan et al., 2018). By analyzing the preferences of sequence context neighboring the targeted “C” on cytosine base editing, we observed that both ancSgo-BE4 and ancSth1a-BE4 displayed significantly decreased editing frequency in the GC context and mostly preferred TC context (TC > CC > AC > GC); in contrast, the bases after the targeted “C” showed nearly no influence on base conversion efficiency (Figures 2C,D).

We also analyzed the by-products induced by ancSgo-BE4 and ancSth1a-BE4 as CBEs. In general, both editors exhibited low frequencies of C-to-non-T conversions (C-to-G or A) and indels (Figures 2E–H), which were comparable with the product purity of the BE4 system in previous reports (Komor et al., 2017; Koblan et al., 2018). Relatively, the indel rate induced by ancSth1a-BE4 was slightly higher than that induced by ancSgo-BE4 in tested sites (Figure 2I). Collectively, ancSgo-BE4 and ancSth1a-BE4 are two robust base editing tools with non-classical editing windows in the non-GC context with low by-products.

### Editing Universality and Optimization of SgoCas9- and Sth1aCas9-Mediated CBEs

**FIGURE 3:**
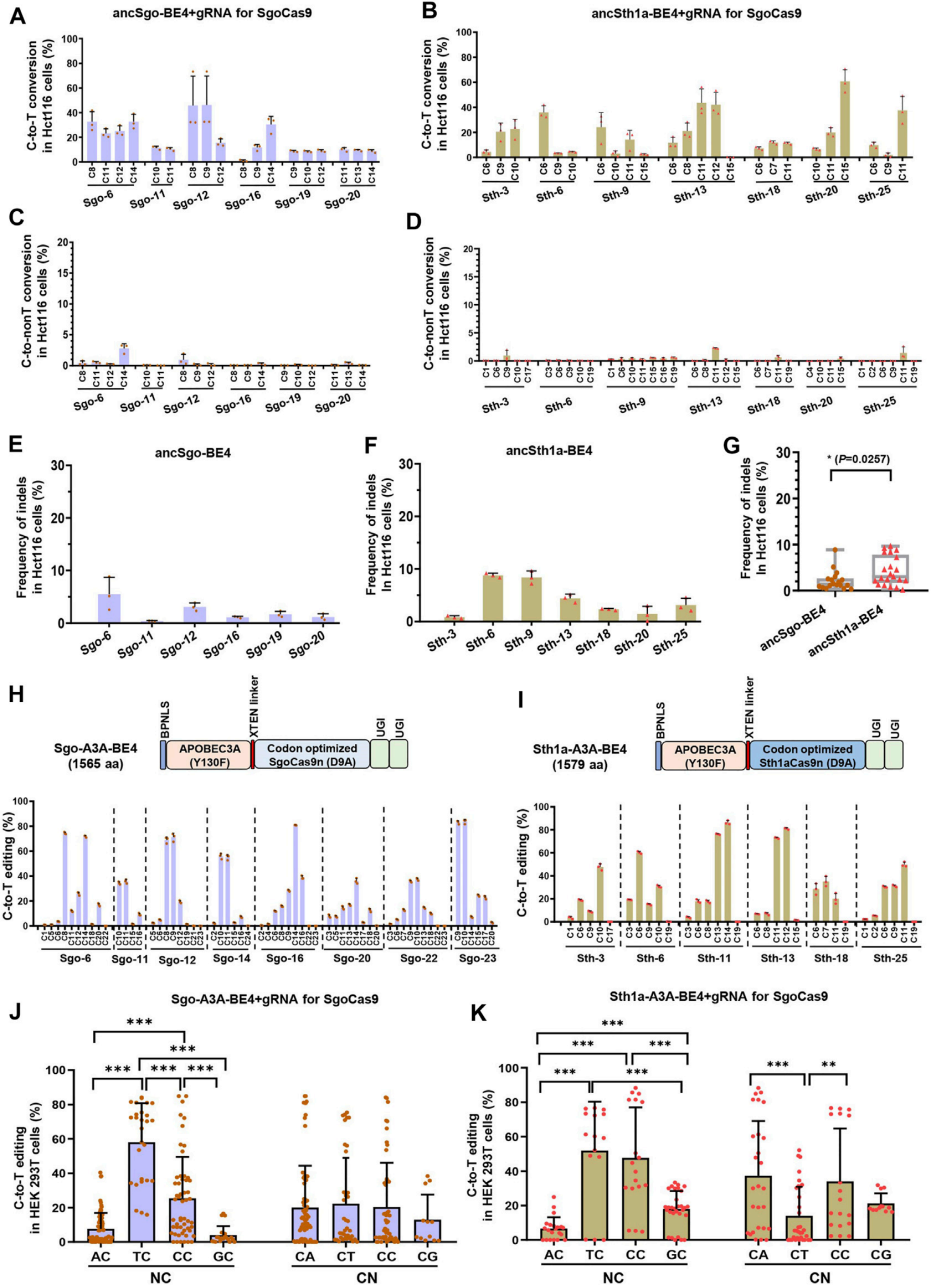
Editing universality of ancSgo-BE4 and ancSth1a-BE4 in human cells and the editing capability of APOBEC3A-mediated CBEs. **(A–F)** The C-to-T conversion efficiencies **(A,B)**, C-to-non-T conversion efficiencies **(C,D)**, and the frequencies of indels **(E,F)** induced by ancSgo-BE4 and ancSth1a-BE4 at targeting sites in Hct116 cells were presented. **(G)** Comparison of frequencies of indels in **E** and **F** from Hct116 cells. *P*-value was calculated from unpaired, two-tailed Student’s t-test (*p < 0.05). **(H,I)** The schematic diagram showing the plasmid architectures of Sgo-A3A-BE4 and Sth1a-A3A-BE4 **(H)** (upper panel) and C-to-T editing efficiencies induced by Sgo-A3A-BE4 and Sth1a-A3A-BE4 in HEK293T cells (lower panel) **(I)**. **(J,K)** Comparison of C-to-T editing activities induced by Sgo-A3A-BE4 **(J)** and Sth1a-A3A-BE4 **(K)** in different neighboring contexts at the 5’ (NC) or 3’ (CN) ends of targeted cytosines (Student’s t-test). **p < 0.01, ***p < 0.001.

To test the universality of ancSgo-BE4 and ancSth1a-BE4, the two CBEs were co-transfected with their gRNAs into Hct116 cells, a colon cancer cell line. We found that ancSgo-BE4 and ancSth1a-BE4 can also induce highly efficient C-to-T base conversions, although the targeting efficiencies were relatively lower than that in HEK293T cells (Figures 3A,B; Figure 1D). Moreover, both ancSgo-BE4 and ancSth1a-BE4 induced low proportions of C-to-non-T conversions and indels (Figures 3C–F), consistent with the observations in HEK293T cells (Figure 2). Similarly, the indel frequencies induced by ancSth1a-BE4 were slightly higher than those induced by ancSgo-BE4 (Figure 3G), which was consistent with the observations in HEK293T cells (Figure 2I).

Considering the observation that both ancSgo-BE4 and ancSth1a-BE4 were inefficient in editing cytosines in the GC context (Figure 2), which will largely restrict the applications of the two CBEs, we tried to optimize the two CBEs by replacing the cytosine deaminase ancAPOBEC1 with engineered human APOBEC3A (Y130F), which has been reported to be able to efficiently deaminate GC and methylated C in various sequence contexts (Wang et al., 2018). Subsequently, the editing capability of the two engineered CBEs, Sgo-A3A-BE4 and Sth1a-A3A-BE4, was determined in HEK293T cells. Both human APOBEC3A (Y130F)-conjugated CBEs displayed high C-to-T conversion efficiencies up to 84.9% (Sgo-A3A-BE4) and 88.1% (Sth1a-A3A-BE4), respectively (Figures 3H,I), which seem to be slightly higher than those displayed by ancSgo-BE4 and ancSth1a-BE4 (Figure 1D). As expected, the inefficient editing in GC contexts induced by ancSgo-BE4 and ancSth1a-BE4 was improved by our optimization with human APOBEC3A deaminase, especially for Sth1aCas9-based CBEs, although they still showed sequence preferences for TC and CC contexts within protospacers (Figures 3J,K; Figures 2C,D). Intriguingly, the C-to-T editing efficiency induced by Sth1a-A3A-BE4 in the GC context was even higher than that in the AC context (Figure 3K).

Meanwhile, the frequencies of C-to-non-T conversions and indels induced by Sgo-A3A-BE4 and Sth1a-A3A-BE4 were generally comparable to those induced by ancSgo-BE4 and ancSth1a-BE4 (Supplementary Figures S5A–D), except for site Sth-6. Interestingly, there was no significant difference for indel rates induced by Sgo-A3A-BE4 and Sth1a-A3A-BE4 (*p* = 0.54), while the average indel rates induced by Sgo-A3A-BE4 (5.7%) were higher than those induced by ancSgo-BE4 (5.7 vs. 2.3%, *p* = 0.0003) (Supplementary Figure S5E). It was consistent with a previous report that human APOBEC3A-conjugated CBEs induced higher indel frequencies than the APOBEC1-mediated BE3 system (Wang et al., 2018).

We also determined the editing feasibility of SgoCas9 and Sth1aCas9 in the adenine base editor (ABE) system, and we replaced SpCas9n with SgoCas9 (D9A) and Sth1aCas9 (D9A) in ABEmax expression vector (Koblan et al., 2018) to obtain ABEmax-Sgo and ABEmax-Sth1a (Supplementary Figures S6A,B). Unfortunately, we did not observe apparent A-to-G conversions in 10 tested target sites for both constructed ABEs (Supplementary Figures S6C,D). It is consistent with the notion that the adenine deaminase of the ABE system is not fully compatible with a shorter Cas9 protein (Huang T. P. et al., 2019; Agudelo et al., 2020). Thus, SgoCas9- and Sth1aCas9-mediated base editors are functional as CBEs but not ABEs in human cells, and optimization of the two CBEs with human eAPOBEC3A (Y130F) can partially eliminate the negative effect of the GC context on C-to-T conversions.

### Comparison of Base Editing Efficiency Mediated by ancSgo-BE4 and ancSth1a-BE4 With ancBE4-NG and ancSpRY-BE4

**FIGURE 4:**
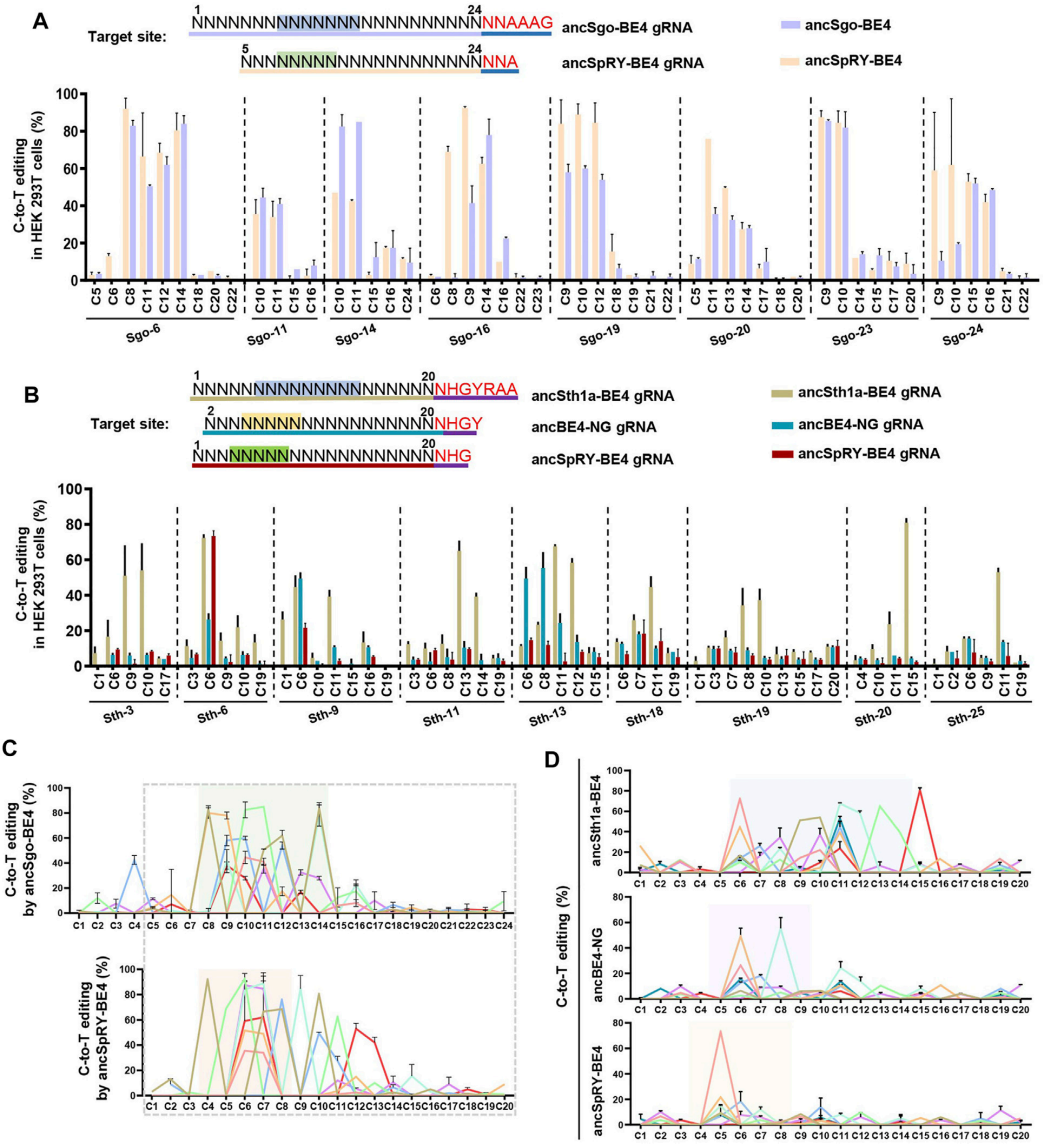
Comparison of ancSgo-BE4 and ancSth1a-BE4 with ancSpRY-BE4 and/or ancBE4-NG. **(A)** Summary of C-to-T editing efficiencies induced by ancSgo-BE4 and ancSpRY-BE4 in HEK293T cells. The upper panel presents the designed target sites with NNAAAG PAM (highlighted in red) to be compatible with SgoCas9 and SpRYCas9 gRNA simultaneously. The most distal nucleotide of ancSgo-BE4 gRNAs relative to NNAAAG within protospacer was set as position “1.” **(B)** Summary of C-to-T editing efficiencies induced by ancSth1a-BE4, ancBE4-NG, and ancSpRY-BE4 in HEK293T cells. The upper panel presents the designed target sites with NHGYRAA PAM (highlighted in red) to be compatible with Sth1aCas9, Cas9-NG (HGY PAM), and SpRYCas9 (NHG PAM) gRNAs simultaneously. The most distal nucleotide of ancSth1a-BE4 gRNA relative to NHGYRAA within protospacer was set as position “1.” **(C,D)** The editing efficiencies from all tested targeting sites were integrated for ancSgo-BE4 and ancSpRY-BE4 **(C)** as well as ancSth1a-BE4, ancBE4-NG, and ancSpRY-BE4 **(D)**, respectively. The dotted box presents the overlapping sequences for their gRNAs, and the colored box highlights the editing window for each CBEs, respectively.

Our results demonstrated that SgoCas9- and Sth1aCas9-mediated CBEs can induce highly efficient C-to-T conversions with NNAAAG and NHGYRAA PAM sequences, respectively. Then, we compared the editing efficiencies of the two base editors with previously reported CBEs, including ancBE4-NG (generated from SpCas9-NG; NGN PAM) and ancSpRY-BE4 (generated from SpRY-Cas9; NNN PAM) (Huang S. et al., 2019; Ren et al., 2021) (Supplementary Figure S4D). Considering the PAM compatibility, ancSgo-BE4 was compared with ancSpRY-BE4 for C-to-T conversions within protospacers with NNAAAG PAM, and the gRNA length was 20-nt for ancSpRY-BE4 (position 5–24 of ancSgo-BE4 gRNA). Overall, the editing efficiency of ancSgo-BE4 resembles that of ancSpRY-BE4 for 8 tested sites, and the individual target site, such as Sgo-14, showed better editing efficiency (Figure 4A). Furthermore, ancSth1a-BE4 was compared with ancBE4-NG and ancSpRY-BE4 for C-to-T conversions for 9 targeting sites with NHGYRAA PAM sequences. The gRNA sequences for ancSth1a-BE4 were identical to those for ancSpRY-BE4 gRNA with 20-nt length. To make the target sequences compatible with ancBE4-NG, the ancBE4-NG gRNAs were constructed with 1-nt shift backward to PAM sequences to generate HGY PAM. Notably, the editing efficiencies of ancSth1a-BE4 were much higher than those of ancSpRY-BE4 and ancBE4-NG for nearly all tested sites (Figure 4B).

We then analyzed the editing windows of the above CBEs under the two groups of comparisons. It showed that the editing window of ancSgo-BE4 (position 8–14) was nearly overlapping with that of ancSpRY-BE4 (position 4–8) because ancSpRY-BE4 gRNAs were 4-nt shorter than ancSgo-BE4 gRNAs (Figure 4C). However, the editing window of ancSth1a-BE4 (position 6–14) was much wider than those of ancBE4-NG and ancSpRY-BE4 (position 4–8) (Figure 4D). Therefore, both ancSgo-BE4 and ancSth1a-BE4 are more suitable for inducing C-to-T conversions within the middle position of protospacers, and ancSth1a-BE4 is applicable for inducing a wide range of C-to-T edits. Considering the editing features, windows, and PAM conditions, ancSgo-BE4 and ancSth1a-BE4 can be added to the toolbox of base editors as key candidate tools.

### Targeting Sequence Preference for ancSgo-BE4 and ancSth1a-BE4

**FIGURE 5:**
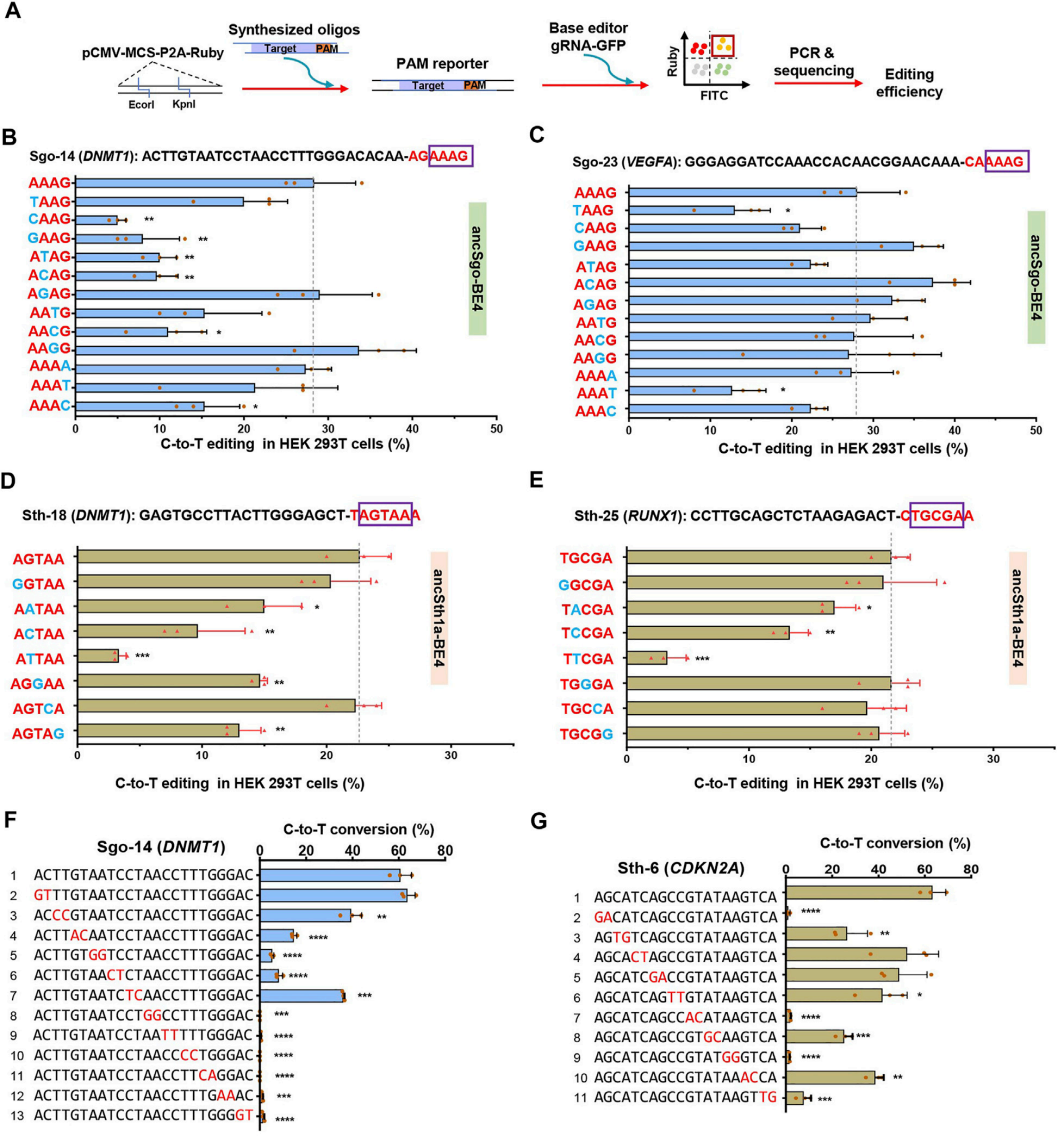
Analysis of editing specificity with mismatches within PAM or target sequences. **(A)** Schematic of strategy for constructing PAM reporter and workflow for testing the effects of PAM mismatches on editing efficiencies. **(B,C)** C-to-T editing efficiencies induced by ancSgo-BE4 in HEK293T cells were presented when ancSgo-BE4 and gRNAs targeting *DNMT1* site Sgo-14 **(B)** and *VEGFA* site Sgo-23 **(C)** were co-transfected with PAM reporters with indicated mismatches (in blue). **(D,E)** C-to-T editing efficiencies induced by ancSth1a-BE4 in HEK293T cells were presented when ancSth1a-BE4 and gRNAs targeting *DNMT1* site Sth-1 **(D)** and *RUNX1* site Sth1a-25 **(E)** were co-transfected with PAM reporters with indicated mismatches (in blue). **(F,G)** Data showing the C-to-T editing efficiencies induced by ancSgo-BE4 **(F)** or ancSth1a-BE4 **(G)** when targeting Sgo-14 or Sth1a-6 with gRNAs constructed with two dinucleotide mismatches (highlighted in red). All editing efficiencies in this figure were calculated from target deep sequencing data (Student’s *t*-test). *p < 0.05, **p < 0.01, ***p < 0.001, ****p < 0.0001.

To further explore the targeting sequence preference for ancSgo-BE4 and ancSth1a-BE4, we constructed a reporter containing an mRuby fluorescent cassette. Synthesized target sequences containing protospacers and PAM sequences were annealed and ligated into the linearized reporter, and mismatched nucleotides can be easily introduced into the reporter. Then, the reporters, base editors, and corresponding gRNAs with a GFP indicator were co-transfected into HEK293T cells, and Ruby/GFP double-positive cells were collected for PCR amplification, sequencing, and editing efficiency determination (Figure 5A).

For ancSgo-BE4-mediated base editing, we mutated one nucleotide of conserved “AAAG” PAM sequence, and the editing efficiencies were examined for targeting Sgo-12 and Sgo-23 in reporter assays. Taking the results from two sites together, we found that the first “A-to-T or C” and the last “G-to-T or C” mutations showed the greatest inhibitory effect on C-to-T conversions, and the middle two “A” showed a preference for nucleotide “G” (Figures 5B,C). Meanwhile, considering the sequence logo of the PAM sequence for Sth1aCas9 with only a conserved nucleotide “G” within “NHGYRAA” (Supplementary Figure S2C), we mutated this “G” within “HGYRA” into C, T, or A, and other nucleotides were mutated into another purine or pyrimidine. Similarly, “G-to-T or C” mutations showed a greater inhibitory effect on C-to-T editing in both tested sites (Figures 5D,E), while other mutations did not significantly influence base editing efficiencies. Because of the limitation of the small number of tested sites, the detailed PAM preference will be systematically identified in our future study.

Next, we evaluated the tolerance for gRNA mismatches during ancSgo-BE4- or ancSth1a-BE4-induced cytosine base editing. We generated a panel of gRNAs with dinucleotide mutations for ancSgo-BE4 and ancSth1a-BE4, respectively. It showed that the dinucleotide mutations near the 5′ end of the protospacer had none or little impact on the editing efficiency of ancSgo-BE4, while the mutations neighboring the PAM sequence displayed a much greater inhibitory effect on C-to-T conversions, with a few exceptions (Figure 5F). Interestingly, the dinucleotide mismatches within ancSth1a-BE4 gRNA sequences that showed remarkable inhibitory effect on editing efficiencies were mainly located in the middle nucleotides within protospacers, and the dinucleotides at the 5′- or 3′-end of gRNA sequences exhibited very weak effects on their on-targeting activities (Figure 5G). Taking two groups of results together, we found that the dinucleotide mismatches containing “C-to-T” interchanges had a greater effect on C-to-T conversions, whereas “A-to-G” interchanges sometimes showed a much weaker effect on their on-targeting activities (Figures 5F,G). The limitation of using this reporter system could not reveal the real editing frequencies in endogenous sites and could only reflect a stringent tendency for PAM recognition and gRNA targeting. Our data indicate that ancSgo-BE4 and ancSth1a-BE4 may recognize less rigid PAM sequences.

### Evaluation of DNA and RNA Off-Targeting Activities Induced by ancSgo-BE4 and ancSth1a-BE4

**FIGURE 6:**
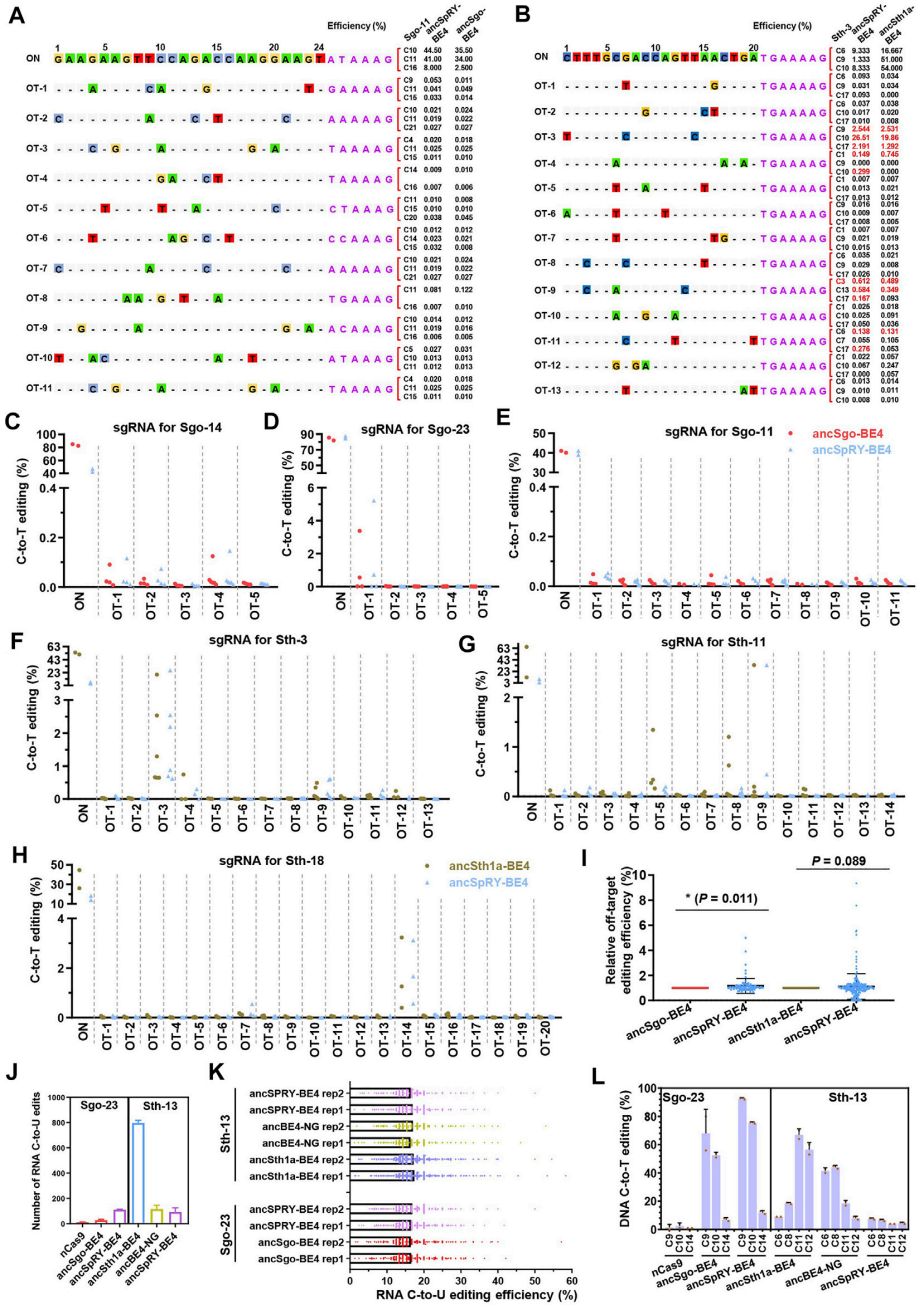
Characterization of DNA or RNA off-targeting features induced by ancSgo-BE4 and ancSth1a-BE4. **(A)** The on-targeting (ON) and off-targeting (OT) C-to-T conversion rates induced by ancSgo-BE4 and ancSpRY-BE4 were presented for two or three cytosines with the highest efficiencies at target site Sgo-13. A total of 11 off-target sites were detected. **(B)** The on-targeting (ON) and off-targeting (OT) C-to-T conversion rates induced by anSth1a-BE4 and ancSpRY-BE4 were presented for two or three cytosines with the highest efficiencies at target site Sth-3. A total of 13 off-target sites were detected. **(C–E)** Comparison of C-to-T off-targeting efficiencies at the on-target site Sgo-14 **(C)**, Sgo-23 **(D)**, and Sgo-11 **(E)** between ancSgo-BE4 and ancSpRY-BE4 in HEK293T cells. **(F–H)** Comparison of C-to-T off-targeting efficiencies at the on-target site Sth-3 **(F)**, Sth-11 **(G)**, and Sth-18 **(H)** between ancSth1a-BE4 and ancSpRY-BE4 in HEK293T cells. Comparison of off-targeting efficiencies in two comparing groups, ancSgo-BE4 vs. ancSpRY-BE4 and ancSth1a-BE4 vs. ancSpRY-BE4. The C-to-T converting efficiencies at the same cytosine were normalized to “1” for ancSgo-BE4 or ancSth1a-BE4. *P*-value was calculated from unpaired, two-tailed Student’s *t*-test. *p < 0.05. **(J)** The number of RNA C-to-U edits in HEK293T cells induced by ancSgo-BE4, ancSth1a-BE4, angBE4-NG, and ancSpRY-BE4 in HEK293T cells was presented. nCas9 served as a negative control, and mock HEK293T sample was used as a control for deducting the naturally occurring C-to-U editing. **(K)** Distributions of RNA C-to-U edits with editing efficiencies induced by ancSgo-BE4, ancSth1a-BE4, ancBE4-NG, and ancSpRY-BE4 in HEK293T cells were presented. **(L)** DNA C-to-T editing efficiency induced by the above CBEs at target sites Sgo-23 and Sth-13 for RNA off-targeting analysis.

All CRISPR-based genome editing tools have the off-targeting potential to operate on DNA in a Cas9-dependent or independent manner, and engineered Cas9 variants and its orthologs may show distinct efficiency, specificity, fidelity, and gRNA compatibility (Rees and Liu, 2018). Therefore, we further investigated the off-targeting activities of our newly generated base editors. Three targeting sites were chosen for on-targeting and off-targeting analyses for ancSgo-BE4 (Sgo-11, Sgo-14, and Sgo-23) and ancSth1a-BE4 (Sth-3, Sth-11, and Sth-18), respectively. A Cas-OFFinder3 online tool in CRISPR RGEN tools was used to predict the potential off-target sites, and the mismatch was set as ≤ 5 for ancSgo-BE4 target sites and ≤3 for ancSth1a-BE4 target sites. These predicted off-target sites were PCR-amplified and subjected to targeted deep sequencing. Accompanying with highly efficient on-target editing induced by ancSgo-BE4, among 20 tested off-target sites in total, only one site (off-target site for Sgo-23) with apparent C-to-T editing was observed (<0.2% for other sites), and off-targeting for this site was also detected with slightly higher efficiency for the ancSpRY-BE4-transfected group (Figures 6A,C–E; Supplementary Figures S7A,B).

For ancSth1a-BE4-targeting groups, a total of 47 off-target sites were detected, and a total of 5 sites with apparent off-target editing (>1%) were observed. Similarly, the off-target C-to-T editing on these sites was also induced by ancSpRY-BE4 (Figures 6B,F–H; Supplementary Figures S7C,D). To compare the off-targeting activities of ancSgo-BE4 and ancSth1a-BE4 with ancSpRY-BE4, the off-targeting efficiency was normalized to “1” for all detected sites for ancSgo-BE4- and ancSth1a-BE4-transfected groups. It demonstrated that the off-targeting efficiency of ancSgo-BE4 was significantly lower than that of ancSpRY-BE4 (*p* = 0.011), while there was no remarkable difference between ancSth1a-BE4 and ancSpRY-BE4 (*p* = 0.089), although with a higher tendency for ancSpRY-BE4 (Figure 6I).

Another aspect of the base editor is deaminase-catalyzed RNA off-targeting activity, which has been largely optimized by engineering or replacement of deaminases (Zhou et al., 2019; Zuo et al., 2020; Li et al., 2021; Wang et al., 2021). Thus, the number of C-to-U RNA edits induced by base editors was analyzed by bulk RNA-seq analysis, and nCas9 served as a control. Notably, only dozens or hundreds of RNA C-to-U edits were detected in HEK293T cells transfected with ancSgo-BE4, ancSth1a-BE4, ancSpRY-BE4, or ancBE4-NG, which were much fewer than that induced by rat APOBEC1-conjugated BE3 with tens of thousands of C-to-U edits (Grunewald et al., 2019) (Figures 6J,K). Relatively, ancSth1a-BE4 induced about 800 RNA C-to-U edits, a bit more than the other three CBE-induced RNA off-target edits, and the number of RNA C-to-U edits was not correlated with their on-targeting activities, especially for Sth-13 targeted by ancSpRY-BE4 with low efficiency (Supplementary Figure S6L). We assume that the extremely low efficiency of the RNA off-targeting activity of these detected CBEs is elicited by ancestral sequence reconstruction of APOBEC1. Ancestral APOBEC1, which lacks 2 phenylalanine residues and an insert of 4 amino acid residues (SITW) across the active site of ancestral deaminases, may preferentially act on DNA but not RNA substrates (Navaratnam and Sarwar, 2006).

### Generation of Pathogenic C-to-T Mutant Mice Using ancSgo-BE4 and ancSth1a-BE4

**FIGURE 7:**
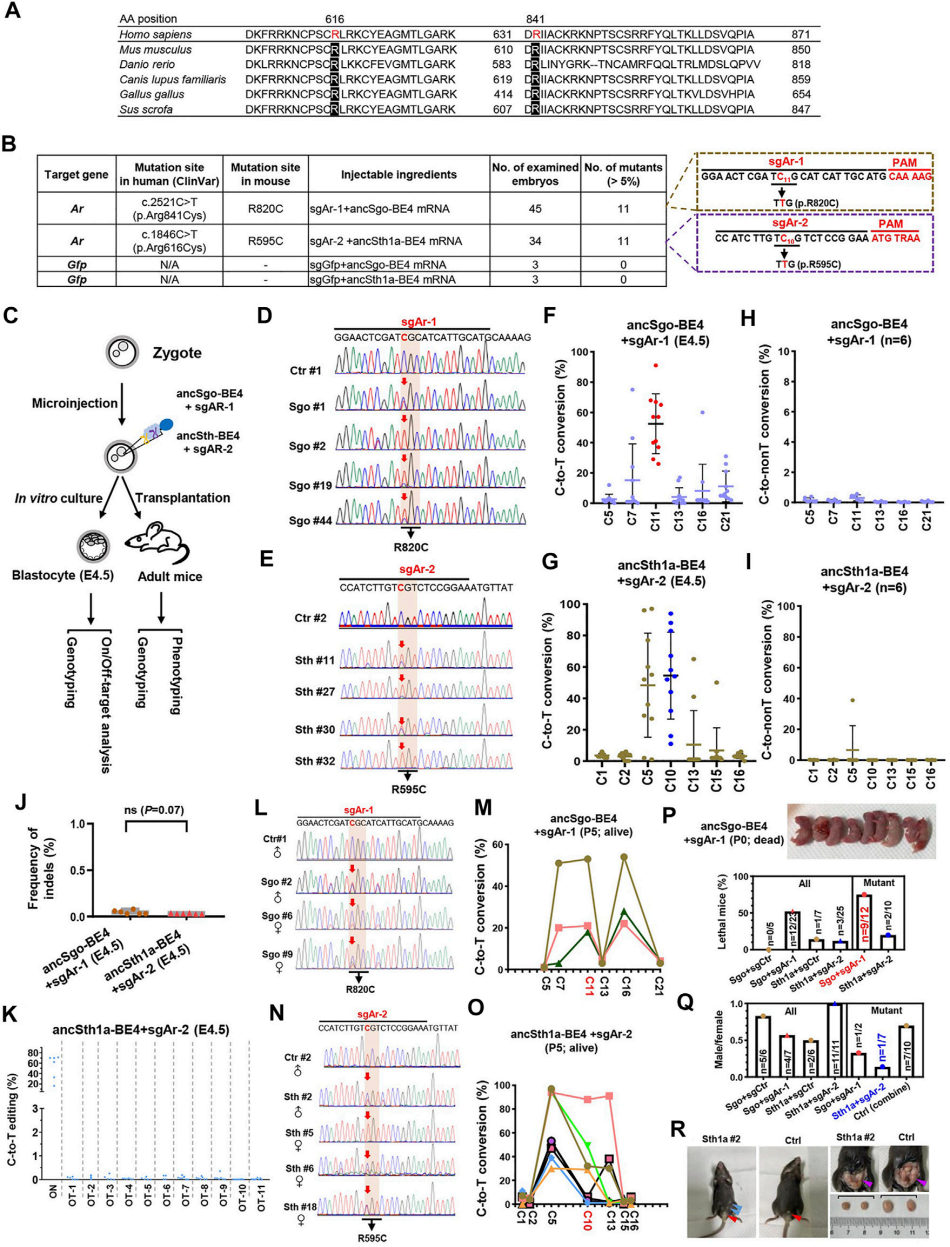
Disease modeling using ancSgo-BE4 and ancSth1a-BE4 in mice. **(A)** Alignment of potential human pathogenic sites (R616 and R841) in Ar protein sequences among multiple organisms. **(B)** Summary of gRNA information targeting R820 and R595 in mouse *Ar* gene. Moreover, the injected reagents, number of examined embryos, and number of mutants with expected mutations (>5% in Sanger sequencing results; < 5% was defined as a non-edited embryo) were presented. Control: sgGFP and ancSgo-BE4 or ancSth1a-BE4. **(C)** Schematic diagram of induction of *Ar* gene mutations using ancSgo-BE4 or ancSth1a-BE4. Meanwhile, genotyping and phenotyping analyses were performed in E4.5 blastocysts from *in vitro* culture and adult mice from transplantation. **(D,E)** Representative sequencing chromatograms from four edited blastocysts using ancSgo-BE4 **(D)** or ancSth1a-BE4 **(E)**. Ctrl, control. Targeted C-to-T conversions were highlighted in red arrows. The codon mutations at target sites are presented under the DNA sequences. **(F,G)** The C-to-T conversion efficiencies of edited mouse E4.5 blastocysts at gRNA targeting sites using ancSgo-BE4 **(F)** and ancSth1a-BE4 **(G)** system. The editing efficiencies of targeted cytosine are highlighted in red **(F)** or blue **(G)** dots. **(H,I)** The C-to-non-T conversion rates of edited mouse E4.5 blastocysts at gRNA targeting sites using ancSgo-BE4 **(H)** and ancSth1a-BE4 **(I)** system. **(J)** The frequency of indels induced by ancSgo-BE4 and ancSth1a-BE4 system in edited mouse E4.5 blastocysts (n = 6 for both groups). *P*-value was calculated from unpaired, two-tailed Student’s *t*-test. **(K)** The C-to-T editing efficiencies at the on-targeting and 11 potential off-targeting sites induced by the ancSth1a-BE4 system in edited mouse E4.5 blastocysts. **(L,O)** Representative sequencing chromatograms from edited mice induced by ancSgo-BE4 **(L)** and ancSth1a-BE4 **(O)** system. **(M,P)** Line charts showing C-to-T conversion in live edited mice using ancSgo-BE4 **(M)** and ancSth1a-BE4 **(P)** system. Each line represents a single mouse with different cytosines within the protospacer. **(N)** The quantities of lethal mice in all or mutant mice at P0. The image on the upper panel shows the dead mice from the ancSgo-BE4-treated group. **(Q)** The ratio between males and females in all or mutant mice. Sgo, ancSgo-BE4; Sth1a, ancSth1a-BE4. **(R)** Sex reversal in founder mice. Left: a 5-week-old mouse (Sth1a #2) with female genitalia (red arrowhead) and nipples (blue arrowheads); middle: control male with normal male genitalia (red arrowhead); right: founder Sth1a #2 with smaller testes (purple arrowhead).

Given that ancSgo-BE4 and ancSth1a-BE4 can efficiently induce C-to-T editing in cultured cells, we are curious about whether ancSgo-BE4 and ancSth1a-BE4 can induce C-to-T conversions *in vivo*. Considering the finding that androgen receptor (*Ar*) is known to be associated with androgen insensitivity syndrome (AIS) and that a lot of mutations with unknown functions have been reported in human patients (Radmayr et al., 1997; Lek et al., 2018), we decide to target *Ar* using the two CBEs. By searching for the ClinVar database at the NCBI, we designated R616C and R841C that were conserved across several specimens (Figure 7A), as our targeting sites. Then we designed two gRNAs, sgAr-1 and sgAr-2, which potentially induce R820C (corresponding to human R841C) and R595C (corresponding to human R616C) mutations using ancSgo-BE4 and ancSth1a-BE4, respectively (Figure 7B).

To test the efficiency of targeting pathogenic sites using ancSgo-BE4 and ancSth1a-BE4, we co-transfected ancSgo-BE4 or ancSth1a-BE4 with sgAr-1 or sgAr-2 gRNA, respectively, into mouse neuroblastoma N2a cells. It demonstrated that about 12 and 6% of targeted C-to-T conversions can be induced by ancSgo-BE4 and ancSth1a-BE4, respectively (Supplementary Figures S8A,B). Subsequently, the two CBEs, sgAr-1 and sgAr-2, were transcribed into mRNAs. Two sets of the mixture, ancSgo-BE4 and sgAr-1, as well as ancSth1a-BE4 and sgAr-2, were microinjected into one-cell embryo, and then E4.5 embryos or adult mice were collected for genotyping and phenotyping (Figure 7C). Most of the injected zygotes can develop normally to blastocyst (45 out of 47, 34 out of 45, 21 out of 22, and 7 out 9 for sgAr-1 targeting, sgAr-2 targeting, ancSgo-BE4 control, and ancSth1a-BE4 control, respectively) (Supplementary Figure S8C). Strikingly, ancSgo-BE4 and sgAr-1 induced 29–91% of C-to-T conversions at the target site in 11 embryos (n = 45 in total), and ancSth1a-BE4 and sgAr-2 induced 11–94% of C-to-T conversions at the target site in 11 embryos (n = 34 in total), which were prominently higher than that in N2a cells; no apparent C-to-T editing was observed in control groups (Figures 7D–G; Supplementary Figures S8D,E). In relative terms, ancSgo-BE4 and sgAr-1 combinations induced precise C-to-T conversions at C11 to elicit R820C mutation, with rare neighboring C7 mutations; however, ancSth1a-BE4 and sgAr-2 induced C-to-T conversions at the C10 target site as well as C5 in most edited embryos (Figures 7F,G).

We also performed targeted deep sequencing analysis in 6 edited embryos for each group, showing high on-targeting activities (Supplementary Figures S8F,G) and low frequencies of C-to-non-T conversions and indels, with an accidental C-to-non-T editing in one embryo (Figures 7H–J). Intriguingly, the indel rates (<0.1% for detected sites) were much lower than those detected in HEK293T cells; there was no significant difference for ancSgo-BE4- and ancSth1a-BE4-induced indels (Figures 2, 7J). Then the potential off-target sites for sgAr-1 and sgAr-2 were predicted by Cas-OFFinder3; no potential off-target sites were predicted for sgAr-1 with up to 5 mismatches, and there were dozens or hundreds of potential off-target sites for sgAr-2 with 3–5 mismatches (Supplementary Figure S8H). We amplified 11 potential off-target sites with 3 mismatches within the protospacer (3 sites were not successfully amplified) for analysis, showing that there was no apparent off-target editing detected for sgAr-2, with C-to-T conversions ranging from 0 to 0.35% (Figure 7K; Supplementary Figure S8I).

After successfully approving the feasibility of both CBEs in the blastocyst, the microinjected embryos were transplanted into pseudopregnant mice for obtaining edited mice. In ancSgo-BE4-treated mice, we acquired 11 live mice with only 3 mice edited (18, 21, and 53% at site C11) (Figures 7L,M), and 12 dead mice after birth with 9 mice edited (8 mice with editing efficiency ranging 5–91% and 1 mouse with indels) (Supplementary Figures S8J,K). Moreover, the percentage of lethal mice for ancSgo-BE4-treated mice was much higher than that for control and ancSth1a-BE4-treated mice, with 52.2% of lethal mice among all obtained mice and 75% of lethality among all successfully edited mice (Supplementary Figure S7N). It seemed that the editing efficiency in dead mice was a bit higher than that in live mice, although it was not significant (Supplementary Figure S8L). It suggests that R820 is essential for Ar gene function and that its mutation may lead to lethality.

In ancSth1a-BE4-treated mice, we obtained 22 live mice with 8 mice edited (with editing efficiency ranging from 1 to 88% at site C10) and 3 lethal mice with 2 mice edited (95 and 96% at site C10) (Figures 7O,P; Supplementary Figure S8M). Among alive edited mice, there was only one male mice (n = 1/8, 12.5%), relative to 50% of male pups in all obtained mice in the ancSth1a-BE4-treated group (Supplementary Figure S7Q). This observation is consistent with classical AIS. Interestingly, after the edited mice became adults with sex characteristics, 1 out of 4 ancSgo-BE4-treated male founders (Sgo #2) and 1 out of 11 ancSgo-BE4-treated male founders (Sth1a #2) displayed typical AIS symptom with sex reversal and female external genitalia (Supplementary Figure S7R; Supplementary Figure S8N). Autopsy further showed that the testes of Sth1a #2 mice were much smaller than those of control mice with wild-type genotype (Supplementary Figure S7R). Collectively, these data demonstrate that our newly generated CBEs, ancSgo-BE4 and ancSth1a-BE4, can install C-to-T mutations with high efficiency in early mouse embryos and adult mice, largely facilitating C-to-T mutation-elicited disease modeling. Using the two CBEs, we successfully construct two types of mice containing pathologic C-to-T mutations and identify human R616C and R841C mutations as potential AIS-associated genetic single-nucleotide variations (SNVs).

## Discussion

The invention of base editing tools largely facilitates introducing point mutations into genomes of various organisms (Porto et al., 2020), while the applications for gene therapy *in vivo* are always restricted by PAM compatibility, plasmid size for adeno-associated virus (AAV) packaging, and editing window for targeted editing. In the present study, we successfully construct two CBEs mediated by SgoCas9 and Sth1aCas9, which achieve highly efficient C-to-T conversions in human or mouse cells or mouse embryos with the editing window at the middle region of the protospacer, preference on non-GC context sites, and non-classical PAM recognition (position 8–14 and NNAAAG for SgoCa9; position 6–14 and NHGYRAA for Sth1aCa9) (Gasiunas et al., 2020).

The superiority of our newly generated CBEs mainly includes their non-classical editing window, expanded PAM compatibility, high editing fidelity, smaller size, and high efficiency. We can move the protospacer to place the targeted “C” at the expected position of the editing window with PAM recognition in consideration, to achieve highly efficient or specific targeting. Their preference on non-GC context sites is a double-edged sword (Figure 2), which can be used to avoid editing of non-targeted “C.” Meanwhile, this will also restrict their applications for editing GC context sites, which can be improved by replacing ancAPOBEC1 with human APOBEC3A (Y130F) deaminase (Figures 3H–K).

Until now, several Cas9 orthologs with distinct PAM recognition have been reported, such as SaCas9 (NNGRRT) (Nishimasu et al., 2015), Nme2Cas9 (N4CC) (Edraki et al., 2019), Sth1Cas9 (NNRNAA) (Zhang et al., 2020), xCas9 (NG, GAA, and GAT) (Hu et al., 2018), SpCas9 (NGG) (Hsu et al., 2013), SpCas9-EQR (NGAG), SpCas9-VQR (NGAN and NGCG) (Kleinstiver et al., 2015), SpCas9-NG (Nishimasu et al., 2018), and SpRY-Cas9 (NNN) (Walton et al., 2020). In the present study, we added two Cas9 ortholog-based CBEs with different PAM sequences (NNAAAG for ancSgo-BE4 and NHGYRAA for ancSth1a-BE4) (Figure 1), which augment the tool box of base editors for targeted mutations. Although their PAM sequences have been identified in *in vitro* biochemical assays (Gasiunas et al., 2020), we achieve efficient C-to-T conversions using the two Cas9 nickase-based CBEs in cultured cells and mouse embryos (Figures 1, 7). Moreover, mismatch analyses for PAM sequences demonstrate that the PAM recognition of the two CBEs is not as stringent as classical SpCas9, which can be explained by the flexible compositions and structures of the PI domain (Supplementary Figures S1–S3), and this feature may further expand their targeting scopes.

We also evaluate the purity of C-to-T-converted products induced by ancSgo-BE4 and ancSth1a-BE4, showing that the proportions of indels and C-to-non-T conversions are comparable to those induced by ancBE4-NG and ancSpRY-BE4 (Komor et al., 2017; Koblan et al., 2018) (Figures 2, 3), while they displayed comparable or higher C-to-T-targeting efficiencies (Figure 4). Similar to SpCas9-based BEs, ancSth1a-BE4 employs 20-nt guide sequences, while ancSgo-BE4 employs 24-nt guide sequences, indicating that ancSgo-BE4 may have the potential for greater on-targeting specificity, although the first 4-nt guide nucleotides seem to be non-essential for its on-targeting fidelity (Figure 5F). Moreover, the majority of dinucleotide mutations within the protospacer strikingly inhibit their on-targeting activities (Figures 5F,G). Moreover, both CBEs show a bit lower DNA off-targeting activities and minimal RNA off-targeting activities relative to ancBE4-NG and ancSpRY-BE4 when fused with ancestral evolved APOBEC1 (Figure 6). It also reminds us that the applications of ancestral deaminase might be an effective pathway for reducing RNA off-targeting activities. Taking these data together, we demonstrate that ancSgo-BE4 and ancSth1a-BE4 can induce efficient C-to-T substitutions with high fidelity and low DNA/RNA off-targeting activities. Superimposing their features with smaller size (Figure 1), SgoCas9- and Sth1aCas9-mediated editing tools have the potential to be developed into AAV-based gene therapeutic tools *in vivo*, such as SauriCas9 and Nme2Cas9 (Edraki et al., 2019; Hu et al., 2020).

Finally, ancSgo-BE4 and ancSth1a-BE4 are utilized to induce precise C-to-T substitutions with up to 92 and 97% efficiencies in edited blastocysts or founder mice. Furthermore, pathogenic *Ar* gene mutation-associated AIS phenotype is observed in the two CBE-edited mice, and two genetic SNVs associated with human AIS disease are identified. The disadvantage of the two CBEs as well as the other reported CBEs is that the targeted “C” and the neighboring “C” are always mutated simultaneously, which may cause some trouble for linking genotype and phenotype. The successful construction of a disease mouse model with targeted point mutations using ancSgo-BE4 and ancSth1a-BE4 approves the potential of the two CBE tools for scientific and therapeutic applications *in vitro* and *in vivo*.

## Materials and Methods

### Plasmid Construction

The pGL3-U6-EGFP, ancBE4max, ABEmax, and APOBEC3A (Y130F)-ancBE4max plasmids were gifts from Dr. Xingxu Huang’s lab from Shanghai Tech University. The DNA fragments of codon-optimized SgoCas9 and Sth1aCas9 were synthesized (Genscript Biotech, China) and used for the construction of ancSgo-BE4 and ancSth1a-BE4 with the ClonExpress II One Step Cloning Kit (Vazyme, China). The final construct sequences for ancSgo-BE4 and ancSth1a-BE4 are presented in Supplementary Table S1. Meanwhile, synthesized scaffold sequences (Genscript Biotech, China) for SgoCas9 or Sth1aCas9 gRNAs were cloned into the pGL3-U6-EGFP vector, with the replacement of scaffold sequences for SpCas9. The construct sequences for ancSgo-BE4 and ancSth1a-BE4 gRNA expression vectors are presented in Supplementary Table S2. To construct gRNAs for ancSgo-BE4 and ancSth1a-BE4, the gRNA oligos were annealed with cohesive ends of BsaI enzyme and then linked to the BsaI-digested pGL3-U6-EGFP template with T4 DNA ligase (NEB, #M0202). The oligo sequences used for constructing gRNAs in the present study are listed in Supplementary Table S3. To construct ancSgo-ABEmax, ancSth1a-ABEmax, Sgo-A3A-BE4, and Sth1a-A3A-BE4, we designed primers to replace ancAPOBEC1 in ancSgo-BE4 and ancSth1a-BE4 with TadA/TadA* or human APOBEC3A (Y130F) sequences using the ClonExpress II One Step Cloning Kit (Vazyme, China). The sequences for coding TadA/TadA* and human APOBEC3A (Y130F) are presented in Supplementary Table S2. The primer sequences for constructing ancSgo-BE4, ancSth1a-BE4, ancSgo-ABEmax, ancSth1a-ABEmax, Sgo-A3A-BE4, Sth1a-A3A-BE4, and their gRNA expression vectors are listed in Supplementary Table S4.

### Cell Culture and Transfection

HEK293T and mouse neuroblastoma N2a cells were cultured in DMEM (Gibco) supplemented with 10% fetal bovine serum (Gibco) and 1% penicillin/streptomycin (Gibco) incubated at 37°C in an atmosphere of 5% CO_2_. Hct116 cells (human colon cancer cell) were cultured in the PRIM-1640 medium (Gibco) supplemented with 10% fetal bovine serum (Gibco) and 1% penicillin/streptomycin (Gibco) under the same culture conditions. The cells were seeded onto 24-well plates (Corning) and transfected with 1,500 ng base editors and 500 ng gRNA expression plasmids by EZtrans (Shanghai life iLAB BIO Technology) per well following the manufacturer’s instructions. After transfection for 72 h, the cells were collected and sorted about 10,000 fluorescent-positive cells using flow cytometry for PCR amplification and sequencing.

### Genomic DNA Extraction and PCR Amplification

The harvested cells were treated with 20 μl lysis buffer (10 mM Tris-HCl (pH 8.0), 50 mM KCl, 1.5 mM MgCl_2_, 0.5% Nonidet P-40, 0.5% Tween-20, and 100 μg/ml proteinase K (ThermoFisher Scientific)) under the PCR procedure (68°C for 30 min, 16°C for 2 min, 98°C for 5 min). The lysates were centrifugated at 12,000 rpm for 3 min. Then the supernatants were PCR-amplified with the procedure (95°C for 5 min of pre-degeneration, 35 repeated cycles (95°C for 30 s, 58°C for 30 s, and 72 °C for 20 s), 72°C for 5 min for extension). PCR products were cleaned up and then determined by Sanger sequencing or targeted deep sequencing. The results from Sanger sequencing were uploaded to EditR (https://moriaritylab.shinyapps.io/editr_v10/) for calculating the mutation rates. The primers used for PCR amplification are listed in Supplementary Table S5.

### Targeted Deep Sequencing and Data Analysis

PCR products were purified by the clean-up kit (AXYGEN) and then subjected to library construction and high-throughput sequencing on an Illumina sequencing platform with PE150 mode (Novogene, China). The amplicon sequencing data were analyzed using CRISPResso2 (v.2.0.31) in the batch mode, with parameters “--base_edit --wc -8 --fastq_output --base_editor_output --write_cleaned_report --place_report_in_output_folder.” Editing efficiency was quantified from the “Quantification_window_nucleotide_percentage_table.txt” table. Indels were quantified from the “Alleles_frequency_table_around_sgRNA_*.txt” table. The results including C-to-T conversion rates, C-to-non-T (C-to-G and C-to-A) conversion rates, and indel rates were calculated.

### DNA Off-Targeting Analysis

Potential off-target sites were performed using Cas-OFFinder3 (http://www.rgenome.net/cas-offinder/), with the maximum 3 or 5 mismatches for Sth1a-Cas9 or Sgo-Cas9 gRNAs as indicated. We used Ensembl (https://asia.ensembl.org/index.html) to retrieve 1,000 bp sequences covering these potential off-target sites and designed appropriate primers to amplify the specific sequences. Targeted deep sequencing was performed to test off-targeting efficiencies. The primers used to amplify potential off-targeting sites are listed in Supplementary Table S6.

### RNA Off-Targeting Analysis

HEK293T cells transfected with base editors and corresponding gRNAs (3 μg base editor and 1 μg gRNA in 6 cm dish) were subjected to cell sorting and RNA extraction. A total of 500 ng RNA was subjected to RNA sequencing analysis on an Illumina sequencing platform with PE150 mode (6 G raw data for each sample) (Novogene, China). Two biological replicates were performed for each RNA off-targeting analysis, and non-transfected HEK293T samples served as a control. RNA off-targeting analysis was performed as we previously described (Li et al., 2021). Briefly, sequencing reads were mapped to the human reference genome (hg38) by STAR software (version 2.5.1) and then annotated from GENCODE version v30. After removing duplications, variants were identified by GATK HaplotypeCaller (version 4.1.2) as following procedures: filtration with QD (quality by depth) < 2, verification and quantification of all variants by bam-readcount with parameters -q 20 -b 30. The depth for a given edit is >10x, and these edits were required to have at least 99% of reads supporting the reference allele in wild-type samples (mock HEK293T sample). Finally, only C-to-U edits in transcribed strands were considered for subsequent analysis (Zhang et al., 2020).

### PAM and gRNA Mismatch Test

We constructed a reporter expressing mRuby with an independent cassette. The reporter was digested with EcoRI (NEB, #R3101L) and KpnI (NEB, #R3142L) enzymes for linearization. Synthesized target oligos including gRNAs, PAMs, and cohesive ends with artificial-designed mismatches were annealed and constructed into the linearized vector. HEK293T cells were transfected with 1,500 ng ancSgo-BE4 or ancSth1a-BE4, 500 ng gRNA (with a GFP indicator), and 100 ng target reporter using EZtrans reagents in 24-well plates, and three replicates were performed for each group. The cells obtained from cell sorting of GFP/mRuby double-positive cells using flow cytometry were subjected to lysis and PCR amplifications using Phanta Max SuperFidelity DNA polymerase (Vazyme; P505) as we previously described (Lin et al., 2021). The target deep sequencing results for calculating the editing efficiencies. The synthesized oligos for PAM and gRNA mismatch test and primers used for PCR amplifications are listed in Supplementary Table S7.

### *In Vitro* Transcription

*In vitro* transcription was performed as we previously described (Qiao et al., 2020). The ancSgo-BE4/ancSth1a-BE4 vectors were linearized by BbsI (NEB, #R3539L) and transcribed *in vitro* using the mMESSAGE mMACHINE T7 ULTRA Kit (Life Technologies, AM1345) according to the manufacturer’s protocol. T7-gRNA PCR products (T7 promoter was contained in the primer sequences) were purified and used as the template for *in vitro* transcription (IVT) using the MEGAshortscript T7 Kit (Life Technologies, AM1354). The transcribed products were purified using the MEGA Transcription Clean-up Kit (Life Technologies, AM 1908) according to the manufacturer’s protocols and eluted in RNase-free water. Primers used for IVT are listed in Supplementary Table S8.

### Microinjection of One-Cell Embryo and Embryo Transfer

Female C57BL/6 mice (4-week-old) were superovulated and mated to C57BL/6 male mice. Zygotes were collected from oviducts of female mice. mRNA mixtures (100 ng/μl ancSgo-BE4/ancSth1a-BE4 and 50 ng/μl gRNA) were injected into the cytoplasm of zygotes in a droplet of the M2 medium containing 5 μg/ml cytochalasin B using a piezo (Primetech) microinjector. The injected zygotes were cultured in the KSOM medium at 37°C under 5% CO_2_ in air. For analyzing editing efficiency in blastocysts, E4.5 embryos were subjected to whole-genome random amplification. Briefly, a single blastocyst was transferred to 5 μl of alkaline lysis solution (200 mM KOH/50 mM dithiothreitol). After incubation for 10 min at 65°C, 5 μl of neutralization solution (900 mM Tris-HCl with pH 8.3, 300 mM KCl/200 mM HCl) was added. Then the lysis was added with 5 μl random primers (400 μM; Genscript, Nanjing, China), 6 μl 10 × PCR buffer (Takara, Dalian, China), 3 μl dNTPs (2.5 mM), 1 μl Taq polymerase (Takara, Dalian, China), and 35 μl ddH_2_O. 50 primer-extension cycles were carried out with the following PCR program: denaturation at 92°C for 1 min, annealing at 37°C for 2 min, ramping step of 10 s/degree to 55°C, and 4 min incubation at 55°C for polymerase extension. Then the products were used as PCR templates. For analyzing editing efficiency in newborn or adult mice, microinjected embryos were transferred to oviducts of pseudopregnant ICR females at 0.5 days post-copulation; tails were collected from newborn or adult mice. Genomic DNA from embryo tails was extracted by using the One Step Mouse Genotyping Kit (Vazyme, PD101). Amplified genomic DNA or DNA from tails was subjected to PCR with Phanta Max Super-Fidelity DNA polymerase (Vazyme; P505). Oligos synthesized for constructing gRNAs and primers used for detecting targeting efficiencies in blastocysts or mice are listed in Supplementary Table S3.

### Phylogenetic Tree Construction, Sequence Alignment, and Structural Prediction

Amino acid sequence of SpCas9 was used as a query in BLAST searches to find all Cas9 orthologs. Orthologs with ≥80% similarities, SaCas9, SgoCas9, and Sth1aCas9 were selected as the target objects to construct the phylogenetic tree. First, the sequence homology was compared by MEGA X, and then the phylogenetic tree was constructed by calculating the most appropriate phylogenetic tree model of the selected amino acid sequence. Next, the phylogenetic tree was obtained by retouching FigTree v1.4.3. We then input the amino acid sequences of SpCas9, SgoCas9, and Sth1aCas9 in multiple sequence alignment online module of Clustal X and compare them to obtain the homologous amino acid information of the three Cas9 orthologs. Referring to the functional domain of SpCas9 in the previous report (Anders et al., 2014), we marked the key functional regions of the three Cas9 orthologs and calculated the amino acid sequence homology between different proteins through DNAMAN (version 5.1.0.0; Lynnon Biosoft). The same method was used for the amino acid sequence alignment of Sth1aCas9 and St1Cas9 as well as the scaffold DNA sequence alignment of SgoCas9 and Sth1aCas9.

Predicted structures of SgoCas9 and Sth1aCas9 were generated by I-TASSER (Yang and Zhang, 2015). The amino acid sequences of SgoCas9 and Sth1aCas9 were retrieved in FASTA format and uploaded to the online server I-TASSER (Iterative Threading ASSEmbly Refinement) (https://zhanggroup.org/I-TASSER/) to build a homology model of the target proteins. The structures of SpCas9 and SthCas9 were downloaded from Protein Data Bank (https://www.rcsb.org/), and the 3D modeled structure was validated by the Rasmol 2.7.2.1.1.

### Statistical Analysis

All data were presented as mean ± standard error from three individual determinations for all experiments. Data were analyzed by Student’s *t*-test *via* GraphPad prism software 8.0.1. *p < 0.05, **p < 0.01, ***p < 0.001, ****p < 0.0001.

## Data Availability

The datasets presented in this study can be found in online repositories. The names of the repository/repositories and accession number(s) can be found below: BIOPROJECT PRJNA756638.
