# Supplementary material for: Two Compact Cas9 Ortholog-Based Cytosine Base Editors Expand the DNA Targeting Scope and Applications In Vitro and In Vivo
Source: Front Cell Dev Biol. 2022 Mar 1;10:809922. doi: 10.3389/fcell.2022.809922 (PMC8921874; doi:10.3389/fcell.2022.809922)
Supplement: Supplementary file 1 [file DataSheet1.pdf]

Supplementary Materials

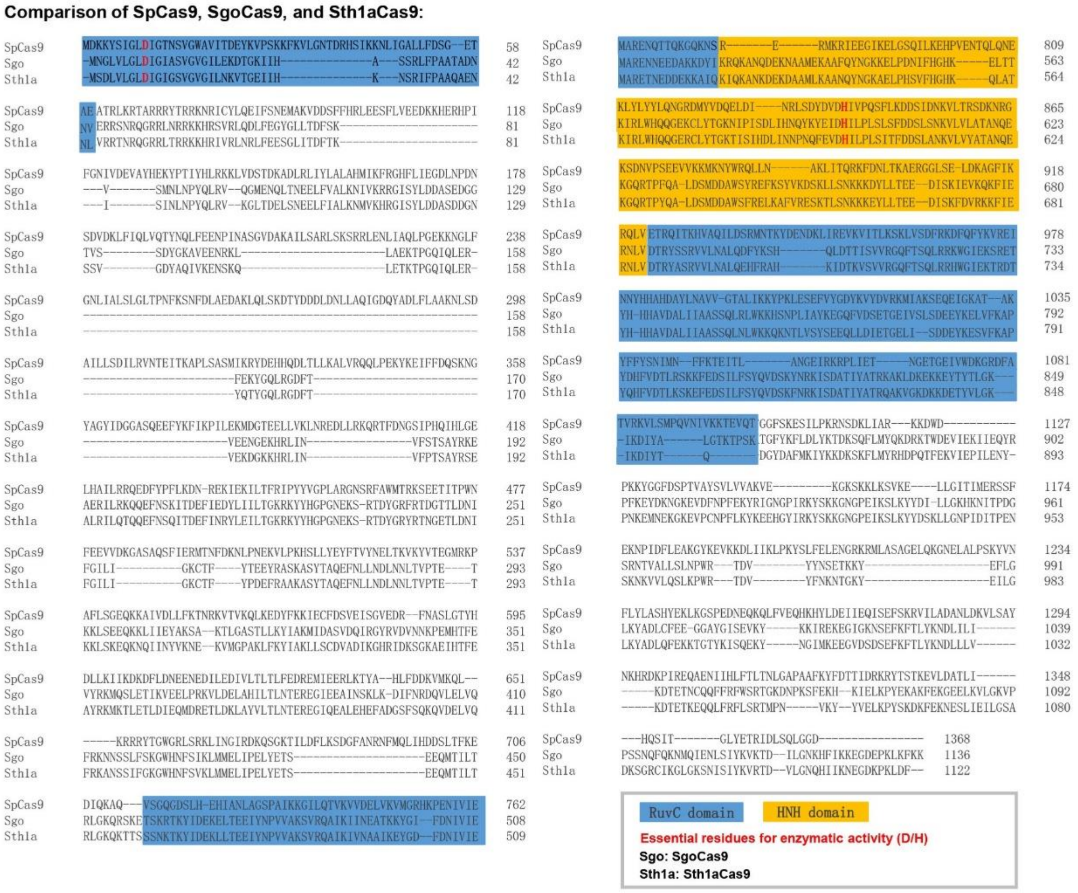

Supplementary Figure 1. Amino acid sequence alignment of SpCas9, SgoCas9, and Sth1aCas9. Two important catalytic domains: RuvC domain (blue) and HNH domain (yellow) are highlighted and the essential residues for enzymatic activity (D/H) are highlighted in red. Sgo, SgoCas9; Sth1a, Sth1aCas9.

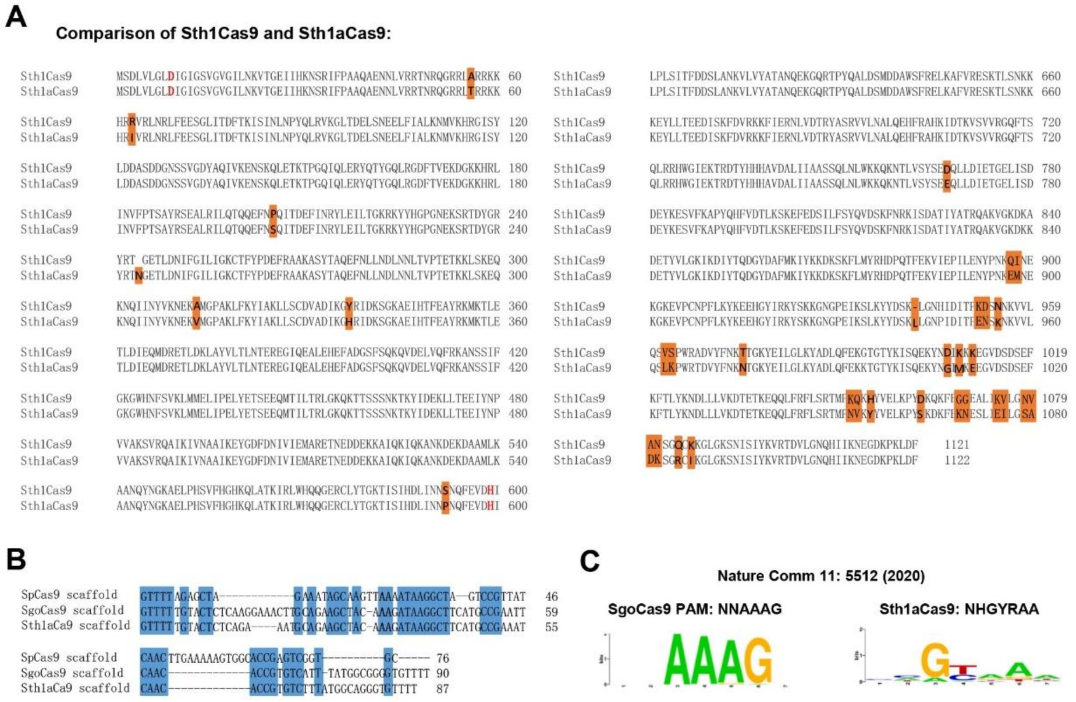

Supplementary Figure 2. Comparison of sequences of Cas9 orthologs and gRNAs.

(**A**) Comparison of Sth1Cas9 and Sth1aCas9 amino acid sequences. The distinct amino acids are highlighted by yellow background.

(**B**) Comparison of the scaffold sequences within gRNAs for SpCas9, SgoCas9, and Sth1aCas9.

(**C**) The PAM sequence logos for SgoCas9 and Sth1aCas9 from Nature Comm 11: 5512 (2020).

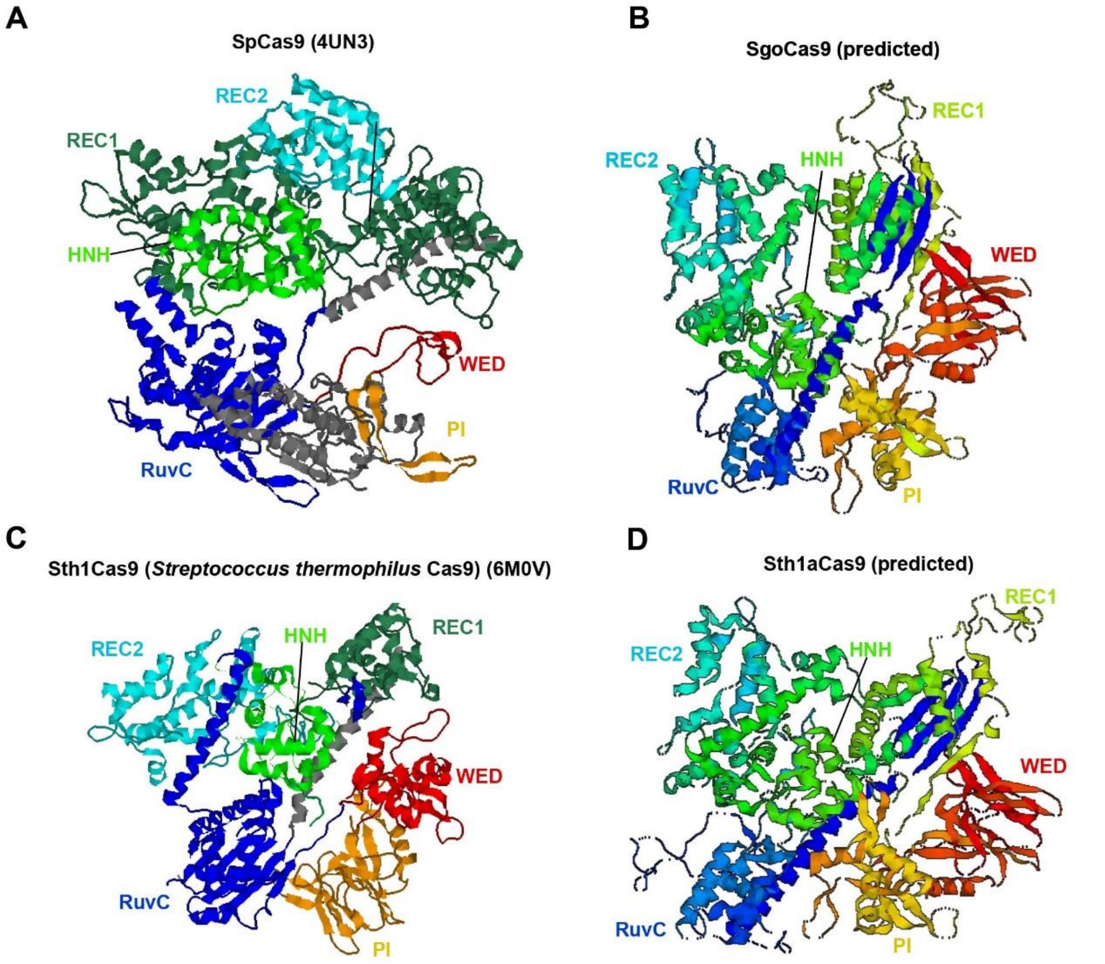

Supplementary Figure 3. Comparison of protein structures of SpCas9 and Sth1Cas9 with predicted structures of SgoCas9 and Sth1aCas9.

(**A-D**) The protein structures of SpCas9 (4UN3) (**A**) and Sth1Cas9 (6M0V) (**C**) were downloaded from Protein Data Bank and generated by Rasmol. The protein structures of SgoCas9 (**B**) and Sth1aCas9 (**D**) were predicted by using I-TASSER and generated by Rasmol. The different domains of Cas9 proteins were labelled in different colors. Similar but not completely same colors were used for labelling same functional domains.

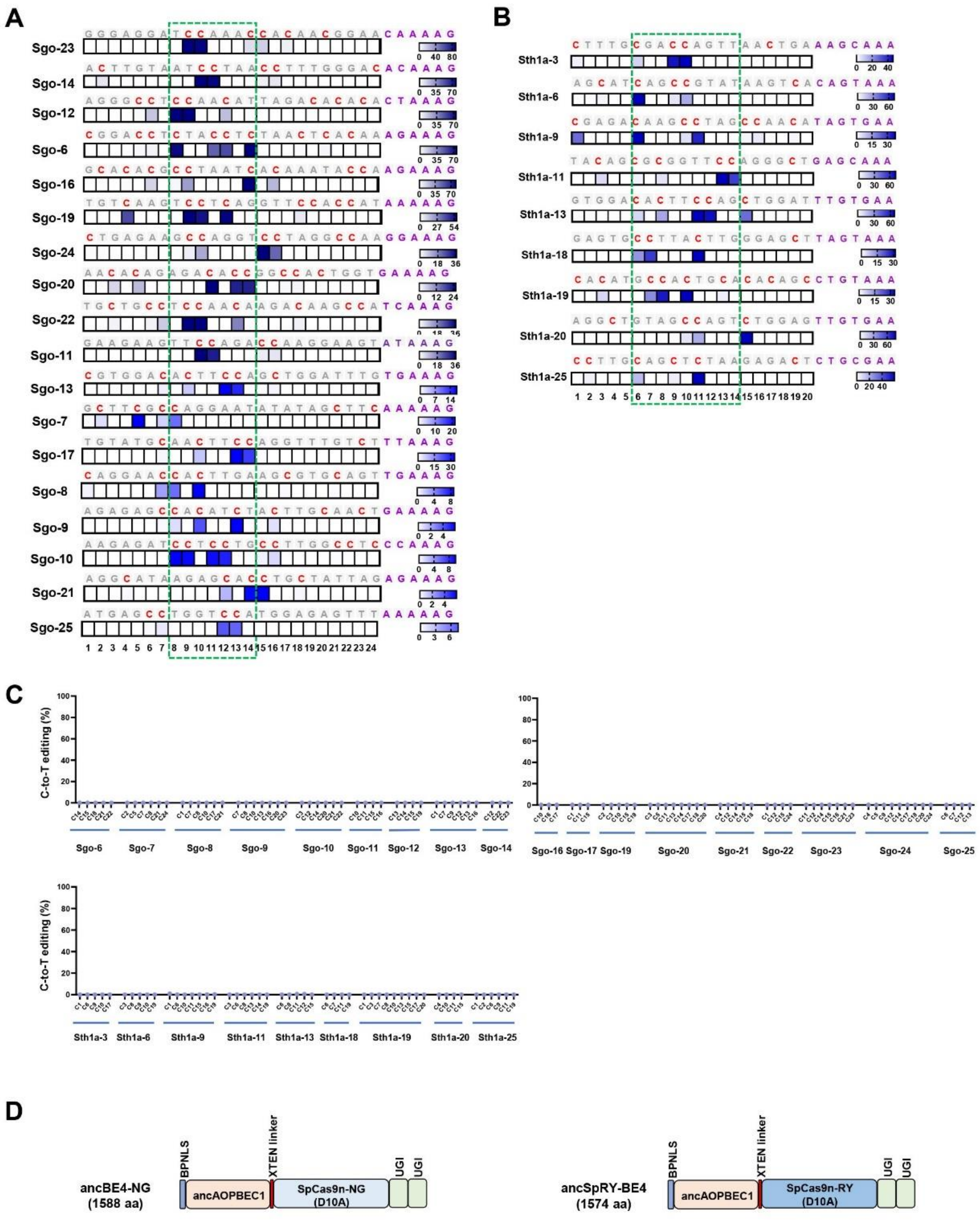

Supplementary Figure 4. C-to-T editing efficiency detection.

(**A** and **B**) The heat map presenting C-to-T editing efficiencies induced by ancSgo-BE4 (**A**) and ancSth1a-BE4 (**B**). Data are presented as mean ± s.d. from three independent experiments. The dashed box shows the editing window for each CBE. The sequences in purple are PAM sequences.

(**C**) The C-to-T editing efficiency at test sites used in the present study wildtype HEK293T cells.

(**D**) Schematic diagram of plasmid architectures for ancBE4-NG and ancSpRY-BE4.

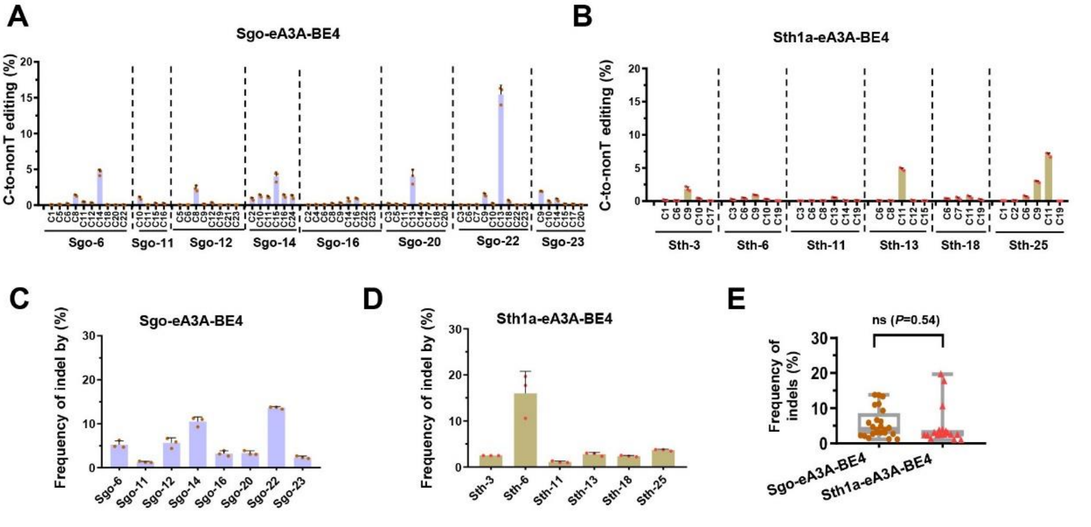

Supplementary Figure 5. C-to-T editing features mediated by human APOBEC3A (Y130F)-mediated CBEs.

(**A** and **B**) The C-to-nonT conversion rates induced by Sgo-A3A-BE4 (**A**) and Sth1a-A3A-BE4 (**B**) in HEK293T cells. The data are generated from targeted deep sequencing analyses from three repeated experiments.

(**C** and **D**) Frequencies of indels induced by Sgo-A3A-BE4 (**C**) and Sth1a-A3A-BE4 (**D**) on detected targeting sites in HEK293T cells.

(**E**) Comparison of indel frequencies induced by Sgo-A3A-BE4 and Sth1a-A3A-BE4 in HEK293T cells.

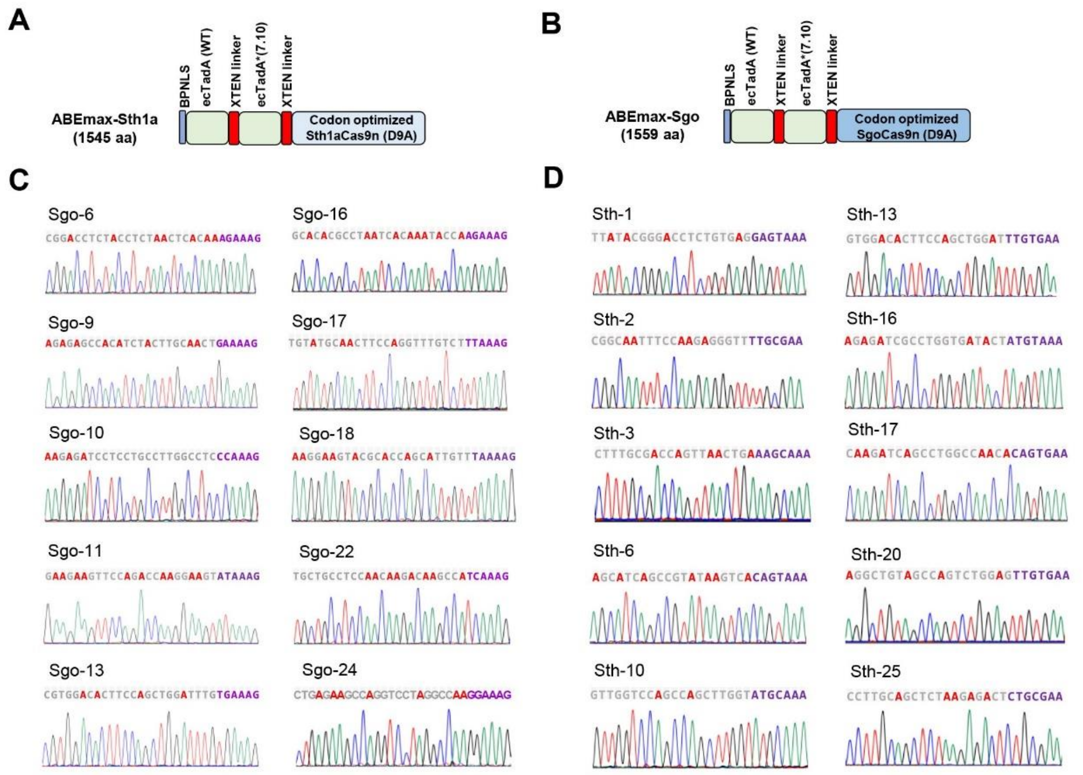

Supplementary Figure 6. SgoCas9- and Sth1aCas9-mediated ABE editing. (**A** and **B**) The schematic diagram of plasmid architectures of ABEmax-Sgo (**A**) and ABEmax-Sth1a (**B**).

(**C**) Sanger sequencing results showing the A-to-G editing ability induced by ABEmax-Sgo (**C**) or ABEmax-Sth1a (**D**) systems in HEK293T cells respectively. 10 target sites were tested for each editor. The sequences in purple are PAM sequences.

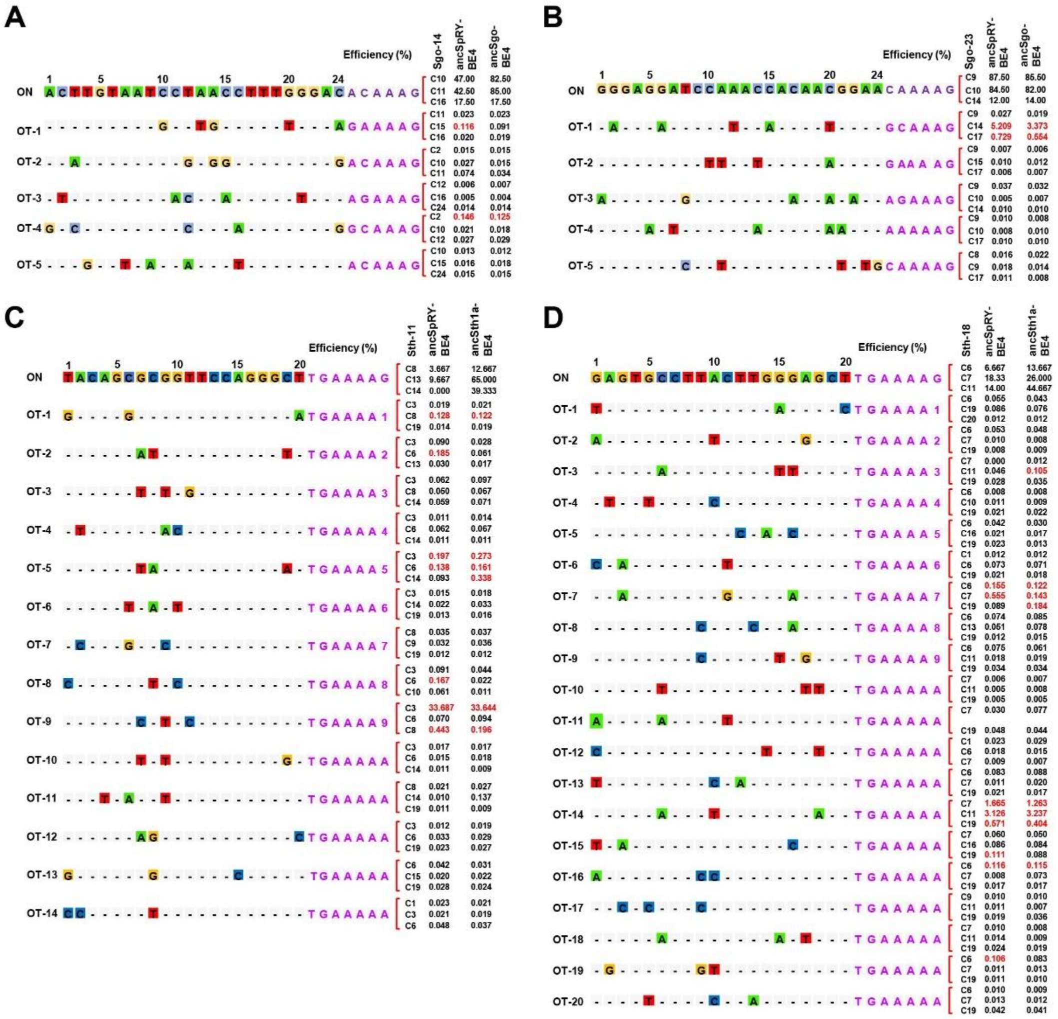

Supplementary Figure 7. DNA off-targeting analysis.

(**A** and **B**) The on-targeting (ON) and off-targeting (OT) C-to-T conversion rates induced by ancSgo-BE4 and ancSpRY-BE4 were presented for three cytosines with highest efficiencies at target site Sgo-14 (**A**) and Sgo-23 (**B**). A total of 5 off-target sites were detected for each on-target site.

(**C** and **D**) The on-targeting (ON) and off-targeting (OT) C-to-T conversion rates induced by ancSth1a-BE4 and ancSpRY-BE4 were presented for two or three cytosines with highest efficiencies at target site Sth-11 and Sth-18. A total of 14 or 20 off-target sites were detected.

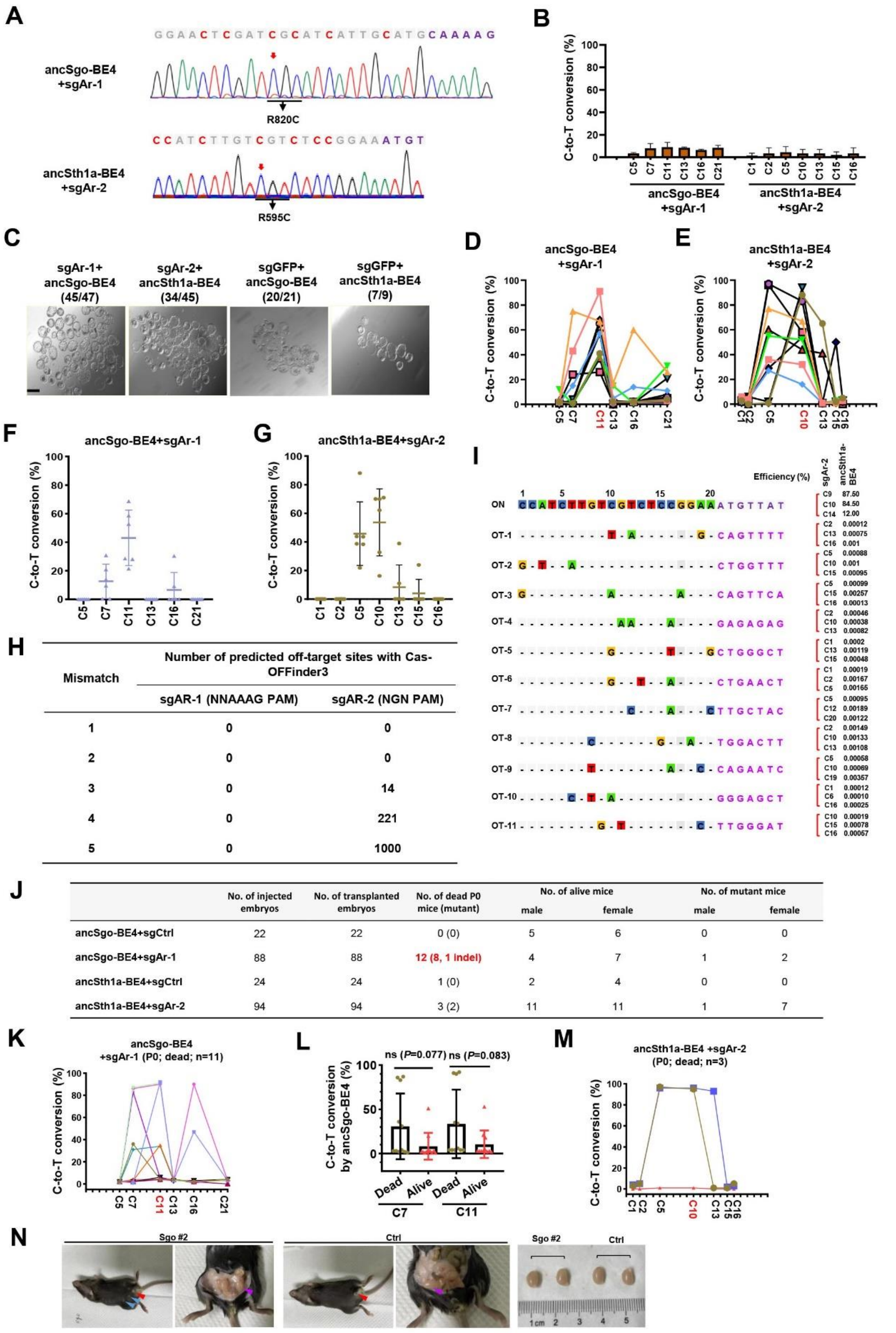

Mismatch	Number of predicted off-target sites with Cas-OFFinder3	
	sgAR-1 (NNAAAG PAM)	sgAR-2 (NGN PAM)
1	0	0
2	0	0
3	0	14
4	0	221
5	0	1000

	No. of injected embryos	No. of transplanted embryos	No. of dead P0 mice (mutant)	No. of alive mice		No. of mutant mice	
				male	female	male	female
ancSgo-BE4+sgCtrl	22	22	0 (0)	5	6	0	0
ancSgo-BE4+sgAr-1	88	88	12 (8, 1 indel)	4	7	1	2
ancSth1a-BE4+sgCtrl	24	24	1 (0)	2	4	0	0
ancSth1a-BE4+sgAr-2	94	94	3 (2)	11	11	1	7

Supplementary Figure 8. Supporting data for ancSgo-BE4- and ancSth1a-BE4-mediated C-to-T editing *in vivo*.

(**A**) Representative Sanger sequencing results showing ancSgo-BE4- and

ancSth1a-BE4-induced C-to-T editing in N2a cells.

(**B**) Statistical analysis of C-to-T editing efficiencies induced by ancSgo-BE4 and ancSth1a-BE4 in N2a cells from three repeated experiments.

(**C**) Images displaying the morphology of microinjected E4.5 embryos. The numbers of all embryos and normal blastocysts were presented.

(**D** and **E**) The C-to-T editing efficiencies induced by ancSgo-BE4 system and sgAr-1 (n=11) (**D**) as well as ancSth1a-BE4 and sgAr-2 (n=11) (**E**) from analyses of Sanger sequencing results. The targeted cytosines were highlighted in red. Each line represents the efficiencies for all cytosines within the protospacer in a single edited blastocyst.

(**F** and **G**) The C-to-T editing efficiencies induced by ancSgo-BE4 system and sgAr-1 (n=6) (**F**) as well as ancSth1a-BE4 and sgAr-2 (n=6) (**G**) from targeted deep sequencing analysis.

(**H**) The number of predicted off-target sites with Cas-OFFinder3.

(**I**) The on-targeting (ON) and off-targeting (OT) C-to-T conversion rates induced by ancSth1a-BE4 were presented for three cytosines with highest efficiencies at the on-target site of sgAr-2. A total of 11 off-target sites with 3 mismatches were detected (3 off-target sites were not successfully amplified).

(**J**) Statistics of injected embryos and obtained mice.

(**K**) The C-to-T editing efficiencies of ancSgo-BE4 system-treated mice that were lethal at P0 (n=11). The mouse with apparent indels was not presented.

(**L**) Comparison of C-to-T conversion efficiencies in dead or live mice that were induced by ancSgo-BE4 and sgAr-1.

(**M**) The C-to-T editing efficiencies of ancSth1a-BE4 system-treated mice that were lethal at P0 (n=3).

(**N**) A 5-week-old mouse (Sgo #2) with female genitalia (red arrowhead) and nipples (blue arrowheads). A control (Ctrl) male mouse with normal male genitalia (red arrowhead). The image that presented the morphology of testes in control and Sgo #2 mice are shown in the right panel.

Supplementary Table 1. The final construct sequences for ancSgo-BE4 and ancSth1a-BE4.

**ancSgo-BE4 (CMV enhancer - CMV promoter-SV40 NLS - ancAPOBEC1 - 32aa linker - nCas9(D9A) - 2*UGI-SV40 NLS - bGH poly(A) signal – ori – AmpR - Amp promoter)**
gacattgattattgactagttattaatagtaatcaattacggggtcattagttcatagcccatatatggagttccgcgttacataacttacggtaaatg<br>gcccgcctggctgaccgcccaacgacccccgcccattgacgtcaataatgacgtatgttcccatagtaacgccaatagggactttccattgac<br>gtcaatgggtggagtatttacggtaaactgcccacttggcagtacatcaagtgtatcatatgccaagtacgccccctattgacgtcaatgacggt<br>aaatggcccgcctggcattatgcccagtacatgaccttatgggactttcctacttggcagtacatctacgtattagtcatcgctattaccatggtgat<br>gcggttttggcagtacatcaatgggcgtggatagcggtttgactcacggggatttccaagtctccaccccattgacgtcaatgggagtttgttttgg<br>caccaaaatcaacgggactttccaaaatgtcgtaacaactccgccccattgacgcaaatgggcggtaggcgtgtacggtgggaggtctatat<br>aagcagagctggtttagtgaaccgtcagatccgctagagatccgcggccgctaatacgactcactatagggagagccgccaccatgaaac<br>ggacagccgacggaagcgagttcgagtcaccaaagaagaagcggaaagtcagcagtgaaaccggaccagtggcagtggacccaacc<br>ctgaggagacggattgagccccatgaatttgaagtgttctttgacccaagggagctgaggaaggagacatgcctgctgtacgagatcaagtg<br>gggcacaagccacaagatctggcgccacagctccaagaacaccacaaagcacgtggaagtgaatttcatcgagaagtttacctccgagc<br>ggcacttctgcccctctaccagctgttccatcacatggtttctgtcttggagcccttgcggcgagtgttccaaggccatcaccgagttcctgtctcag<br>caccctaacgtgaccctggtcatctacgtggcccggctgtatcaccacatggaccagcagaacaggcagggcctgcgcgatctggtgaattc<br>tggcgtgaccatccagatcatgacagccccagagtacgactattgctggcggaacttcgtgaattatccacctggcaaggaggcacactggc<br>caagatacccacccctgtggatgaagctgtatgcactggagctgcacgcaggaatcctgggcctgcctccatgtctgaatatcctgcggaga<br>aagcagccccagctgacatttttcaccattgctctgcagtcttgtcactatcagcggctgcctcctcatattctgtgggctacaggcctgaagtctg<br>gaggatctagcggaggatcctctggcagcgagacaccaggaacaagcgagtcagcaacaccagagagcagtggcggcagcagcggc<br>ggcagcAACGGCCTGGTGCTGGGCCTGGCCATCGGCATCGCCTCTGTGGGCGTGGGCATCCTGG<br>AGAAAGACACTGGCAAGATCATTCACGCTTCGAGCAGACTGTTCCCAGCCGCCACAGCCGACAA<br>CAATGTGGAGAGACGGAGCAATAGACAGGGCAGACGGCTAAACCGGCGGAAAAAGCACAGATC<br>CGTGCGGCTGCAGGACCTGTTTGAAGGATACGGCCTGCTGACAGACTTCAGCAAGGTGTCCAT<br>GAACCTGAATCCCTACCAGCTGCGGGTGCAGGGAATGGAAAACCAGCTGACCAACGAGGAGCT<br>GTTCGTGGCCCTGAAGAATATCGTGAAGAGAAGAGGCATCAGCTACCTGGACGATGCCAGCGAG<br>GACGGCGGCACCGTGAGTAGCGACTACGGCAAGGCTGTGGAAGAAAACAGAAAACTGCTGGCG<br>GAAAAGACGCCCGGCCAAATCCAGCTGGAACGCTTCGAGAAGTATGGCCAGCTGAGAGGCGAC<br>TTCACCGTGGAAGAAAATGGCGAGAAGCATAGACTGATCAACGTGTTCAGCACCAGCGCCTACA<br>GAAAGGAAGCTGAACGGATCCTGCGGAAGCAGCAGGAGTTCAACAGCAAGATCACAGACGAGT<br>TTATTGAGGACTACCTGATCATCCTGACAGGAAAACGGAAGTACTACCACGGACCTGGCAACGAG<br>AAGAGCAGAACCGACTACGGCAGATTCAGAACCGACGGCACCACCCTGGACAACATCTTCGGC<br>ATCCTGATTGGAAAGTGTACATTCTACACCGAAGAGTATCGGGCCTCTAAGGCCAGCTACACAGC<br>CCAGGAGTTCAACCTGCTCAACGATCTGAACAACCTGACCGTGCCTACCGAGACAAAGAAACTG<br>AGCGAGGAGCAGAAGAAGCTGATCATCGAGTACGCCAAATCTGCCAAGACCCTCGGCGCCAGC<br>ACCCTGCTGAAATATATCGCCAAAATGATCGACGCCAGCGTCGACCAGATCAGAGGCTACCGGG<br>TGGACGTGAACAACAAGCCCGAGATGCACACCTTCGAGGTCTACCGAAAGATGCAGAGCCTGG<br>AAACAATCAAGGTGGAAGAACTGCCTAGAAAGGTCCTGGATGAACTGGCCCACATCCTCACCCT<br>GAATACCGAGAGAGAGGGCATCGAGGAGGCCATCAACAGCAAGCTGAAGGACATCTTCAACCG<br>CGACCAGGTGCTGGAGCTGGTGCAGTTCAGAAAGAACAACAGCAGTCTGTTCTCCAAGGGATG<br>GCACAACTTCAGCATCAAGCTGATGATGGAACTGATCCCAGAGCTGTATGAAACATCCGAAGAAC<br>AGATGACCATCCTGACAAGACTGGGCAAACAGCGTTCTAAGGAGACCTCTAAGCGGACCAAATA<br>CATCGATGAGAAAGAACTGACCGAGGAGATCTATAACCCCGTGGTGGCCAAAAGCGTCCGGCAG

GCCATCAAGATCATCAACGAGGCCACTAAGAAGTACGGCATTTTCGACAACATCGTGATCGAGAT
GGCCAGAGAAAACAACGAAGAAGATGCCAAGAAAGATTATATTAAAAGGCAAAAAGCTAATCAAG
ATGAAAAGAACGCCGCCATGGAAAAGGCTGCATTCCAGTACAATGGCAAGAAGGAACTGCCTGA
TAATATCTTTCACGGCCACAAGGAGCTGACAACAAAAATTCGGCTGTGGCACCAGCAGGGAGAA
AAGTGCCTGTACACCGGAAAGAATATCCCTATCTCTGATCTTATTCACAACCAGTACAAGTACGAG
ATCGACCACATCCTGCCCCTGTCCCTGAGCTTTGACGACTCTCTGAGCAACAAGGTTCTGGTTCT
GGCCACCGCCAACCAGGAGAAGGGCCAAAGAACTCCTTTCCAGGCCCTGGACAGCATGGACGA
CGCCTGGAGCTACAGAGAGTTCAAGAGCTACGTGAAAGACTCTAAACTGCTGTCTAACAAGAAG
AAAGACTACCTGTTGACAGAGGAGGATATCTCCAAGATCGAGGTCAAGCAGAAATTCATCGAGAG
AAATCTGGTGGATACCAGATACAGCTCCAGAGTGGTTCTGAATGCCCTTCAAGACTTCTACAAGA
GCCACCAGCTGGACACCACCATCTCAGTGGTGCGGGGCCAGTTTACCAGCCAGCTGCGGAGAA
AGTGGGGCATCGAGAAAAGCAGGGAAACCTACCACCACCATGCCGTAGACGCTCTTATCATTGC
TGCCTCTAGCCAGCTGCGGCTGTGGAAGAAGCACAGCAACCCTCTGATCGCCTATAAGGAGGGC
CAGTTTGTGGACAGCGAGACAGGCGAGATCGTGTCTCTGTCCGACGAAGAATACAAGGAACTGG
TGTTTAAGGCCCCTTACGATCACTTTGTGGATACCCTGAGAAGCAAGAAATTCGAAGATAGCATCC
TGTTTAGCTATCAAGTGGATTCTAAGTACAACAGAAAGATCTCCGATGCAACAATCTACGCGACCA
GGAAGGCTAAGCTGGATAAGGAAAAGAAGGAGTACACATACACCCTCGGAAAGATCAAAGATATC
TACGCCCTGGGCACAAAGACCCCTTCCAAGACCGGATTCTACAAGTTCCTGGACCTGTACAAGA
CCGATAAGAGCCAGTTCCTGATGTACCAAAAGGATAGAAAGACCTGGGACGAGGTGATCGAGAA
AATCATCGAGCAGTACCGGCCTTTTAAGGAGTACGACAAGAACGGCAAAGAGGTGGATTTCAAC
CCCTTCGAGAAGTACAGAATCGGCAATGGCCCCATCCGGAAATACAGCAAGAAGGGCAACGGAC
CTGAGATCAAGAGTCTGAAATATTACGACATCCTGCTGGGCAAACACAAGAACATCACTCCTGAC
GGATCTAGAAACACCGTGGCCCTGCTGAGCCTGAACCCTTGGAGAACAGACGTGTACTACAACA
GCGAAACAAAGAAGTACGAGTTCCTGGGACTCAAGTACGCCGACCTGTGCTTCGAAGAGGGCG
GAGCCTACGGCATCAGCGAGGTGAAGTACAAGAAGATCAGAGAAAAGGAGGGCATCGGCAAGA
ATAGCGAGTTCAAGTTCACCCTGTACAAGAACGACCTGATTCTGATCAAGGACACCGAAACCAAC
TGCCAGCAGTTCTTCAGATTCTGGAGCAGAACCGGTAAGGACAACCCTAAATCTTTCGAAAAGCA
TAAGATCGAGCTGAAGCCTTACGAGAAAGCCAAGTTCGAGAAAGGCGAGGAGCTAAAAGTGCTG
GGCAAGGTGCCACCTTCTTCCAACCAGTTTCAGAAGAACATGCAAATCGAGAACTTGAGCATCTA
CAAGGTCAAGACAGACATCCTGGGTAACAAACACTTTATCAAAAAGGAGGGAGATGAACCCAAG
CTCAAGTTCAAGAAGagcggcgggagcggcgggagcggggggagcactaatctgagcgacatcattgagaaggagactggg
aaacagctggtcattcaggagtccatcctgatgctgcctgaggaggtggaggaagtgatcggcaacaagccagagtctgacatcctggtgc
acaccgcctacgacgagtccacagatgagaatgtgatgctgctgacctctgacgcccccgagtataagccttgggccctggtcatccaggatt
ctaacggcgagaataagatcaagatgctgagcggaggatccggaggatctggaggcagcaccaacctgtctgacatcatcgagaaggag
acaggcaagcagctggtcatccaggagagcatcctgatgctgcccgaagaagtcgaagaagtgatcggaaacaagcctgagagcgatat
cctggtccataccgcctacgacgagagtaccgacgaaaatgtgatgctgctgacatccgacgccccagagtataagccctgggctctggtca
tccaggattccaacggagagaacaaaatcaaaatgctgtctggcggctcaaaaagaaccgccgacggcagcgaattcgagcccaagaa
gaagaggaaagtctaaccggtcatcatcaccatcaccattgagtttaaacccgctgatcagcctcgactgtgccttctagttgccagccatctgtt
gtttgcccctcccccgtgccttccttgaccctggaaggtgccactcccactgtcctttcctaataaaatgaggaaattgcatcgcattgtctgagta
ggtgtcattctattctggggggtggggtggggcaggacagcaagggggaggattgggaagacaatagcaggcatgctggggatgcggtgg
gctctatggcttctgaggcggaaagaaccagctggggctcgataccgtcgacctctagctagagcttggcgtaatcatggtcatagctgtttcct
gtgtgaaattgttatccgctcacaattccacacaacatacgagccggaagcataaagtgtaaagcctaggatgcctaatgagtgagctaactc
acattaattgcgttgcgctcactgcccgctttccagtcgggaaacctgtcgtgccagctgcattaatgaatcggccaacgcgcgggaagaggc
ggtttgcgtattgggcgctcttccgcttcctcgctcactgactcgctgcgctcggtcgttcggctgcggcgagcggtatcagctcactcaaaggcg

gtaatacggttatccacagaatcaggggataacgcaggaaagaacatgtgagcaaaaggccagcaaaaggccaggaaccgtaaaaag
gccgcgttgctggcgtttttccataggctccgcccccctgacgagcatcacaaaaatcgacgctcaagtcagaggtggcgaaacccgacag
gactataaagataccaggcgtttccccctggaagctccctcgtgcgctctcctgttccgaccctgccgcttaccggatacctgtccgcctttctccc
ttcgggaagcgtggcgctttctcatagctcacgctgtaggtatctcagttcggtgtaggtcgttcgctccaagctgggctgtgtgcacgaaccccc
cgttcagcccgaccgctgcgccttatccggtaactatcgtcttgagtccaacccggtaagacacgacttatcgccactggcagcagccactggt
aacaggattagcagagcgaggtatgtaggcggtgctacagagttcttgaagtggtggcctaactacggctacactagaagaacagtatttggt
atctgcgctctgctgaagccagttaccttcggaaaaagagttggtagctcttgatccggcaaacaaaccaccgctggtagcggtggtttttttgttt
gcaagcagcagattacgcgcagaaaaaaaggatctcaagaagatcctttgatcttttctacggggtctgacactcagtggaacgaaaactca
cgttaagggattttggtcatgagattatcaaaaaggatcttcacctagatccttttaaattaaaaatgaagttttaaatcaatctaaagtatatatga
gtaaacttggtctgacagttaccaatgcttaatcagtgaggcacctatctcagcgatctgtctatttcgttcatccatagttgcctgactccccgtcgt
gtagataactacgatacgggagggcttaccatctggccccagtgctgcaatgataccgcgagacccacgctcaccggctccagatttatcag
caataaaccagccagccggaagggccgagcgcagaagtggtcctgcaactttatccgcctccatccagtctattaattgttgccgggaagct
agagtaagtagttcgccagttaatagtttgcgcaacgttgttgccattgctacaggcatcgtggtgtcacgctcgtcgtttggtatggcttcattcag
ctccggttcccaacgatcaaggcgagttacatgatcccccatgttgtgcaaaaaagcggttagctccttcggtcctccgatcgttgtcagaagta
agttggccgcagtgttatcactcatggttatggcagcactgcataattctcttactgtcatgccatccgtaagatgcttttctgtgactggtgagtactc
aaccaagtcattctgagaatagtgtatgcggcgaccgagttgctcttgcccggcgtcaatacgggataataccgcgccacatagcagaacttt
aaaagtgctcatcattggaaaacgttcttcggggcgaaaactctcaaggatcttaccgctgttgagatccagttcgatgtaacccactcgtgcac
ccaactgatcttcagcatcttttactttcaccagcgtttctgggtgagcaaaaacaggaaggcaaaatgccgcaaaaaagggaataagggcg
acacggaaatgttgaatactcatactcttcctttttcaatattattgaagcatttatcagggttattgtctcatgagcggatacatatttgaatgtatttag
aaaaataaacaaataggggttccgcgcacatttccccgaaaagtgccacctgacgtcgacggatcgggagatcgatctcccgatcccctag
ggtcgactctcagtacaatctgctctgatgccgcatagttaagccagtatctgctccctgcttgtgtgttggaggtcgctgagtagtgcgcgagca
aaatttaagctacaacaaggcaaggcttgaccgacaattgcatgaagaatctgcttagggttaggcgttttgcgctgcttcgcgatgtacgggc
cagatatacgcgtt

ancSth1a-BE4 (CMV enhancer - CMV promoter - SV40 NLS - ancAPOBEC1 - 32aa linker - nCas9(D9A) - 10aa linker - 2*UGI - 14aa linker - SV40 NLS - bGH poly(A) signal – ori – AmpR - Amp promoter)

gacattgattattgactagttattaatagtaatcaattacggggtcattagttcatagcccatatatggagttccgcgttacataacttacggtaaatg
gcccgcctggctgaccgcccaacgacccccgcccattgacgtcaataatgacgtatgttcccatagtaacgccaatagggactttccattgac
gtcaatgggtggagtatttacggtaaactgcccacttggcagtacatcaagtgtatcatatgccaagtacgccccctattgacgtcaatgacggt
aaatggcccgcctggcattatgcccagtacatgaccttatgggactttcctacttggcagtacatctacgtattagtcatcgctattaccatggtgat
gcggttttggcagtacatcaatgggcgtggatagcggtttgactcacggggatttccaagtctccaccccattgacgtcaatgggagtttgttttgg
caccaaaatcaacgggactttccaaaatgtcgtaacaactccgccccattgacgcaaatgggcggtaggcgtgtacggtgggaggtctatat
aagcagagctggtttagtgaaccgtcagatccgctagagatccgcggccgctaatacgactcactatagggagagccgccaccatgaaac
ggacagccgacggaagcgagttcgagtcaccaaagaagaagcggaaagtcagcagtgaaaccggaccagtggcagtggacccaacc
ctgaggagacggattgagccccatgaatttgaagtgttctttgacccaagggagctgaggaaggagacatgcctgctgtacgagatcaagtg
gggcacaagccacaagatctggcgccacagctccaagaacaccacaaagcacgtggaagtgaatttcatcgagaagtttacctccgagc
ggcacttctgcccctctaccagctgttccatcacatggtttctgtcttggagcccttgcggcgagtgttccaaggccatcaccgagttcctgtctcag
caccctaacgtgaccctggtcatctacgtggcccggctgtatcaccacatggaccagcagaacaggcagggcctgcgcgatctggtgaattc
tggcgtgaccatccagatcatgacagccccagagtacgactattgctggcggaacttcgtgaattatccacctggcaaggaggcacactggc
caagatacccacccctgtggatgaagctgtatgcactggagctgcacgcaggaatcctgggcctgcctccatgtctgaatatcctgcggaga
aagcagccccagctgacatttttcaccattgctctgcagtcttgtcactatcagcggctgcctcctcatattctgtgggctacaggcctgaagtctg
gaggatctagcggaggatcctctggcagcgagacaccaggaacaagcgagtcagcaacaccagagagcagtggcggcagcagcggc
ggcagcAGCGACCTGGTTCTGGGCCTAGCTATCGGCATCGGAAGCGTGGGAGTGGGCATCCTGAA
TAAAGTGACCGGCGAGATCATCCACAAGAACAGCAGAATCTTTCCAGCCGCCCAGGCCGAGAAC

AACCTGGTTCGAAGAACCAACAGACAGGGCAGACGGCTGACAAGAAGAAAGAAACACAGGATT
GTGAGACTGAACAGACTGTTCGAGGAGTCCGGCCTGATTACCGACTTCACCAAGATCAGCATCA
ACCTGAACCCCTACCAGCTGCGGGTGAAAGGCCTGACGGATGAGCTGAGCAATGAGGAGCTCT
TCATCGCCCTGAAGAACATGGTGAAGCACCGTGGAATCTCTTACCTGGATGACGCCTCCGACGA
CGGAAACAGCAGCGTGGGCGACTACGCCCAGATCGTGAAGGAAAACAGCAAACAGTTGGAAAC
AAAGACCCCTGGCCAGATCCAGCTGGAAAGATACCAGACATACGGACAGCTGAGGGGCGATTTT
ACCGTGGAAAAGGATGGTAAAAAGCACAGACTGATCAACGTGTTCCCTACCTCCGCCTACCGGA
GCGAGGCCCTGAGAATCCTGCAAACCCAGCAGGAGTTCAACTCTCAGATCACCGATGAGTTCAT
CAACCGGTACCTGGAAATCCTGACCGGCAAAAGAAAGTACTACCACGGCCCAGGCAACGAGAAG
TCTAGAACCGACTACGGCAGATATAGAACCAACGGCGAGACCCTGGATAATATCTTCGGCATCCT
GATCGGAAAGTGCACCTTCTACCCTGACGAGTTTAGAGCCGCTAAGGCCTCTTACACCGCCCAG
GAGTTTAACCTGCTGAACGACCTGAACAACCTGACTGTGCCCACCGAAACCAAGAAGCTCTCTA
AAGAGCAGAAGAACCAAATCATCAACTACGTGAAGAACGAGAAGGTCATGGGTCCCGCCAAGCT
GTTCAAGTATATCGCCAAGCTGCTGTCTTGTGACGTGGCCGACATCAAGGGCCACAGAATCGAC
AAGAGCGGGAAAGCAGAAATCCACACGTTCGAGGCTTACCGGAAGATGAAGACCCTGGAAACC
CTGGACATCGAACAGATGGACAGAGAAACACTGGACAAGCTGGCTTACGTGCTGACCCTGAACA
CCGAAAGAGAAGGAATTCAGGAGGCCCTGGAACACGAGTTCGCCGATGGCAGCTTCTCTCAGA
AACAGGTCGATGAACTGGTGCAGTTCAGAAAAGCCAACTCTAGCATCTTCGGCAAGGGCTGGCA
CAACTTCAGCGTTAAGTTGATGATGGAACTGATCCCCGAACTCTACGAGACAAGCGAGGAGCAA
ATGACCATCCTGACCCGGCTGGGCAAGCAGAAGACGACCAGCTCTTCTAATAAGACAAAGTACAT
CGATGAGAAACTGCTCACAGAGGAAATCTACAACCCAGTGGTGGCCAAGAGCGTCCGGCAGGC
CATCAAGATCGTGAATGCTGCTATCAAGGAATACGGCGACTTCGACAACATCGTGATCGAGATGG
CCAGAGAGACAAACGAGGATGACGAAAAGAAGGCCATCCAAAAAATCCAGAAGGCCAACAAGGA
TGAGAAAGATGCTGCTATGCTGAAGGCTGCCAATCAGTACAACGGAAAAGCTGAGCTGCCCCAC
AGCGTGTTCCACGGCCACAAGCAGCTTGCCACAAAGATTAGACTGTGGCACCAGCAGGGAGAG
AGGTGTCTGTACACCGGAAAGACCATCAGCATCCACGACCTGATCAACAACCCTAACCAGTTTGA
GGTGGACCATATTCTGCCTCTGTCTATCACCTTCGACGATTCTCTGGCCAATAAAGTGCTGGTGTA
CGCTACAGCCAACCAGGAGAAGGGCCAGAGAACACCTTACCAAGCTCTGGACAGCATGGATGA
CGCCTGGTCCTTCAGAGAGCTGAAGGCCTTCGTGCGAGAGAGCAAGACCCTGAGCAATAAGAA
AAAAGAGTACCTGCTGACAGAAGAGGACATCTCCAAGTTCGATGTTCGGAAGAAGTTCATCGAG
CGGAACCTCGTGGACACAAGATACGCCAGCAGAGTGGTGCTGAATGCCCTGCAGGAACACTTC
CGGGCCCACAAGATCGACACCAAAGTGAGCGTGGTGCGGGGACAGTTCACCAGCCAGCTGAGA
CGGCACTGGGGCATCGAGAAGACAAGAGATACCTATCACCACCACGCCGTGGACGCTCTGATCA
TCGCCGCCAGCAGCCAGCTGAACCTGTGGAAGAAACAGAAGAATACCCTGGTCAGCTACTCCGA
GGAACAACTGCTGGACATCGAAACCGGCGAGCTGATCAGCGACGACGAGTACAAGGAGTCCGT
GTTTAAGGCCCCTTATCAGCATTTCGTGGACACACTGAAGTCCAAGGAATTCGAGGACTCTATCC
TGTTCAGCTACCAAGTGGATTCTAAATTCAACAGAAAGATCAGCGACGCCACCATCTACGCCACC
AGACAGGCCAAGGTGGGCAAGGACAAGAAGGACGAAACATACGTGCTGGGCAAGATCAAGGAC
ATCTATACCCAGGACGGCTACGACGCTTTCATGAAGATCTACAAGAAAGACAAGTCCAAGTTCCT
GATGTACAGACATGATCCTCAGACATTTGAGAAGGTGATCGAGCCTATCCTGGAAAACTACCCTAA
CAAGGAGATGAACGAAAAGGGCAAAGAGGTGCCGTGCAACCCCTTCCTGAAATACAAAGAGGAA
CATGGATATATACGGAAATACAGCAAGAAGGGCAATGGCCCTGAAATTAAGAGTCTGAAGTACTAC
GACAGCAAGCTGCTGGGCAACCCTATCGACATCACACCTGAGAACAGCAAGAACAAAGTGGTCC
TGCAGAGCCTGAAGCCTTGGCGGACAGATGTGTACTTCAACAAGAACACCGGCAAGTACGAGAT

CTTGGGCCTCAAGTACGCCGATCTGCAATTTGAGAAGAAAACAGGCACCTACAAGATCAGCCAA
GAGAAGTACAACGGCATCATGAAGGAAGAAGGCGTGGACAGCGACTCCGAATTCAAGTTCACAC
TGTACAAAAACGACCTCCTGCTAGTGAAGGACACCGAGACCAAGGAGCAGCAGCTGTTTCGGTT
CCTGAGCAGGACAATGCCTAACGTGAAGTACTACGTGGAACTGAAGCCCTACAGCAAGGACAAG
TTTGAGAAGAATGAATCCCTGATCGAGATCCTGGGCTCTGCCGACAAGAGCGGCAGATGCATCA
AGGGCCTGGGCAAAAGCAACATCAGCATCTACAAGGTGCGGACCGACGTGTTAGGCAATCAGCA
CATCATAAAGAACGAGGGCGACAAGCCTAAGTTGGATTTCagcggcgggagcggcgggagcggggggagcac
taatctgagcgacatcattgagaaggagactgggaaacagctggtcattcaggagtccatcctgatgctgcctgaggaggtggaggaagtg
atcggcaacaagccagagtctgacatcctggtgcacaccgcctacgacgagtccacagatgagaatgtgatgctgctgacctctgacgccc
ccgagtataagccttgggccctggtcatccaggattctaacggcgagaataagatcaagatgctgagcggaggatccggaggatctggagg
cagcaccaacctgtctgacatcatcgagaaggagacaggcaagcagctggtcatccaggagagcatcctgatgctgcccgaagaagtcg
aagaagtgatcggaaacaagcctgagagcgatatcctggtccataccgcctacgacgagagtaccgacgaaaatgtgatgctgctgacat
ccgacgccccagagtataagccctgggctctggtcatccaggattccaacggagagaacaaaatcaaaatgctgtctggcggctcaaaaa
gaaccgccgacggcagcgaattcgagcccaagaagaagaggaaagtctaaccggtcatcatcaccatcaccattgagtttaaacccgctg
atcagcctcgactgtgccttctagttgccagccatctgttgtttgcccctcccccgtgccttccttgaccctggaaggtgccactcccactgtcctttc
ctaataaaatgaggaaattgcatcgcattgtctgagtaggtgtcattctattctggggggtggggtggggcaggacagcaagggggaggattg
ggaagacaatagcaggcatgctggggatgcggtgggctctatggcttctgaggcggaaagaaccagctggggctcgataccgtcgacctct
agctagagcttggcgtaatcatggtcatagctgtttcctgtgtgaaattgttatccgctcacaattccacacaacatacgagccggaagcataaa
gtgtaaagcctaggatgcctaatgagtgagctaactcacattaattgcgttgcgctcactgcccgctttccagtcgggaaacctgtcgtgccagc
tgcattaatgaatcggccaacgcgcggggaagaggcggtttgcgtattgggcgctcttccgcttcctcgctcactgactcgctgcgctcggtcgttc
ggctgcggcgagcggtatcagctcactcaaaggcggtaatacggttatccacagaatcaggggataacgcaggaaagaacatgtgagca
aaaggccagcaaaaggccaggaaccgtaaaaaggccgcgttgctggcgtttttccataggctccgcccccctgacgagcatcacaaaaat
cgacgctcaagtcagaggtggcgaaacccgacaggactataaagataccaggcgtttccccctggaagctccctcgtgcgctctcctgttcc
gaccctgccgcttaccggatacctgtccgcctttctcccttcgggaagcgtggcgctttctcatagctcacgctgtaggtatctcagttcggtgtag
gtcgttcgctccaagctgggctgtgtgcacgaaccccccgttcagcccgaccgctgcgccttatccggtaactatcgtcttgagtccaacccggt
aagacacgacttatcgccactggcagcagccactggtaacaggattagcagagcgaggtatgtaggcggtgctacagagttcttgaagtggt
ggcctaactacggctacactagaagaacagtatttggtatctgcgctctgctgaagccagttaccttcggaaaaagagttggtagctcttgatcc
ggcaaacaaaccaccgctggtagcggtggtttttttgtttgcaagcagcagattacgcgcagaaaaaaaggatctcaagaagatcctttgatct
tttctacggggtctgacactcagtggaacgaaaactcacgttaagggattttggtcatgagattatcaaaaaggatcttcacctagatccttttaaa
ttaaaaatgaagttttaaatcaatctaaagtatatatgagtaaacttggtctgacagttaccaatgcttaatcagtgaggcacctatctcagcgatc
tgtctatttcgttcatccatagttgcctgactccccgtcgtgtagataactacgatacgggagggcttaccatctggccccagtgctgcaatgatac
cgcgagacccacgctcaccggctccagatttatcagcaataaaccagccagccggaagggccgagcgcagaagtggtcctgcaactttat
ccgcctccatccagtctattaattgttgccgggaagctagagtaagtagttcgccagttaatagtttgcgcaacgttgttgccattgctacaggcat
cgtggtgtcacgctcgtcgtttggtatggcttcattcagctccggttcccaacgatcaaggcgagttacatgatcccccatgttgtgcaaaaaagc
ggttagctccttcggtcctccgatcgttgtcagaagtaagttggccgcagtgttatcactcatggttatggcagcactgcataattctcttactgtcat
gccatccgtaagatgcttttctgtgactggtgagtactcaaccaagtcattctgagaatagtgtatgcggcgaccgagttgctcttgcccggcgtc
aatacgggataataccgcgccacatagcagaactttaaaagtgctcatcattggaaaacgttcttcggggcgaaaactctcaaggatcttacc
gctgttgagatccagttcgatgtaacccactcgtgcacccaactgatcttcagcatcttttactttcaccagcgtttctgggtgagcaaaaacagg
aaggcaaaatgccgcaaaaaagggaataagggcgacacggaaatgttgaatactcatactcttcctttttcaatattattgaagcatttatcag
ggttattgtctcatgagcggatacatatttgaatgtatttagaaaaataaacaaataggggttccgcgcacatttccccgaaaagtgccacctga
cgtcgacggatcgggagatcgatctcccgatcccctagggtcgactctcagtacaatctgctctgatgccgcatagttaagccagtatctgctcc
ctgcttgtgtgttggaggtcgctgagtagtgcgcgagcaaaatttaagctacaacaaggcaaggcttgaccgacaattgcatgaagaatctgct
tagggttaggcgttttgcgctgcttcgcgatgtacgggccagatatacgcgtt

Supplementary Table 2. The construct sequences for ancSgo-BE4 and ancSth1a-BE4 gRNA expression vectors and the sequences for coding TadA/TadA* and human APOBEC3A (Y130F).

Sgo-gRNA vector sequence (U6 promoter - gRNA scaffold - EGFP – ori – AmpR - AmpR promoter)

gagggcctatttcccatgattccttcatatttgcatatacgatacaaggctgttagagagataattggaattaatttgactgtaaacacaaagatatt
agtacaaaatacgtgacgtagaaagtaataatttcttgggtagtttgcagttttaaaattatgttttaaaatggactatcatatgcttaccgtaacttg
aaagtatttcgatttcttggctttatatatcttgtggaaaggacgaaacaccgtgagaccgagagagggtctcaGTTTTTGTACTCTCA
AGGAAACTTGCAGAAGCTACAAAGATAAGGCTTCATGCCGAATTCAACACCCTGTCATTTATGGC
GGGGTGTTTTttttttaaagaattctcgacctcgagacaaatggcagtattcatccacaattttaaaagaaaaggggggattggggggt
acagtgcaggggaaagaatagtagacataatagcaacagacatacaaactaaagaattacaaaaacaaattacaaaaattcaaaattttc
gggtttattacagggacagcagagatccactttggccgcggctcgaggggggttggggttgcgccttttccaaggcagccctgggtttgcgcag
ggacgcggctgctctgggcgtggttccgggaaacgcagcggcgccgaccctgggactcgcacattcttcacgtccgttcgcagcgtcacccg
gatcttcgccgctacccttgtgggccccccggcgacgcttcctgctccgcccctaagtcgggaaggttccttgcggttcgcggcgtgccggacg
tgacaaacggaagccgcacgtctcactagtaccctcgcagacggacagcgccagggagcaatggcagcgcgccgaccgcgatgggctg
tggccaatagcggctgctcagcagggcgcgccgagagcagcggccgggaaggggcggtgcgggaggcggggtgtggggcggtagtgt
gggccctgttcctgcccgcgcggtgttccgcattctgcaagcctccggagcgcacgtcggcagtcggctccctcgttgaccgaatcaccgacc
tctctccccagggggatccatggtgagcaagggcgaggagctgttcaccggggtggtgcccatcctggtcgagctggacggcgacgtaaac
ggccacaagttcagcgtgtccggcgagggcgagggcgatgccacctacggcaagctgaccctgaagttcatctgcaccaccggcaagctg
cccgtgccctggcccaccctcgtgaccaccctgacctacggcgtgcagtgcttcagccgctaccccgaccacatgaagcagcacgacttctt
caagtccgccatgcccgaaggctacgtccaggagcgcaccatcttcttcaaggacgacggcaactacaagacccgcgccgaggtgaagtt
cgagggcgacaccctggtgaaccgcatcgagctgaagggcatcgacttcaaggaggacggcaacatcctggggcacaagctggagtac
aactacaacagccacaacgtctatatcatggccgacaagcagaagaacggcatcaaggtgaacttcaagatccgccacaacatcgagga
cggcagcgtgcagctcgccgaccactaccagcagaacacccccatcggcgacggccccgtgctgctgcccgacaaccactacctgagca
cccagtccgccctgagcaaagaccccaacgagaagcgcgatcacatggtcctgctggagttcgtgaccgccgccgggatcactctcggcat
ggacgagctgtacaagtaaagcggccgcgactctagatcataatcagccataccacatttgtagaggttttacttgctttaaaaaacctcccac
acctccccctgaacctgaaacataaaatgaatgcaattgttgttgttaacttgtttattgcagcttataatggttacaaataaagcaatagcatcac
aaatttcacaaataaagcatttttttcactgcattctagttgtggtttgtccaaactcatcaatgtatcttagtcgaccgatgcccttgagagccttcaa
cccagtcagctccttccggtgggcgcggggcatgactatcgtcgccgcacttatgactgtcttctttatcatgcaactcgtaggacaggtgccgg
cagcgctcttccgcttcctcgctcactgactcgctgcgctcggtcgttcggctgcggcgagcggtatcagctcactcaaaggcggtaatacggtt
atccacagaatcaggggataacgcaggaaagaacatgtgagcaaaaggccagcaaaaggccaggaaccgtaaaaaggccgcgttgct
ggcgttttttccataggctccgcccccctgacgagcatcacaaaaatcgacgctcaagtcagaggtggcgaaacccgacaggactataaag
ataccaggcgtttccccctggaagctccctcgtgcgctctcctgttccgaccctgccgcttaccggatacctgtccgcctttctcccttcgggaagc
gtggcgctttctcatagctcacgctgtaggtatctcagttcggtgtaggtcgttcgctccaagctgggctgtgtgcacgaaccccccgttcagccc
gaccgctgcgccttatccggtaactatcgtcttgagtccaacccggtaagacacgacttatcgccactggcagcagccactggtaacaggatt
agcagagcgaggtatgtaggcggtgctacagagttcttgaagtggtggcctaactacggctacactagaagaacagtatttggtatctgcgctc
tgctgaagccagttaccttcggaaaaagagttggtagctcttgatccggcaaacaaaccaccgctggtagcggtggtttttttgtttgcaagcag
cagattacgcgcagaaaaaaaggatctcaagaagatcctttgatcttttctacggggtctgacgctcagtggaacgaaaactcacgttaaggg
attttggtcatgagattatcaaaaaggatcttcacctagatccttttaaattaaaaatgaagttttaaatcaatctaaagtatatatgagtaaacttgg
tctgacagttaccaatgcttaatcagtgaggcacctatctcagcgatctgtctatttcgttcatccatagttgcctgactccccgtcgtgtagataact
acgatacgggagggcttaccatctggccccagtgctgcaatgataccgcgggacccacgctcaccggctccagatttatcagcaataaacc
agccagccggaagggccgagcgcagaagtggtcctgcaactttatccgcctccatccagtctattaattgttgccgggaagctagagtaagta
gttcgccagttaatagtttgcgcaacgttgttgccattgctacaggcatcgtggtgtcacgctcgtcgtttggtatggcttcattcagctccggttccc
aacgatcaaggcgagttacatgatcccccatgttgtgcaaaaaagcggttagctccttcggtcctccgatcgttgtcagaagtaagttggccgc

agtgttatcactcatggttatggcagcactgcataattctcttactgtcatgccatccgtaagatgcttttctgtgactggtgagtactcaaccaagtc
attctgagaatagtgtatgcggcgaccgagttgctcttgcccggcgtcaatacgggataataccgcgccacatagcagaactttaaaagtgctc
atcattggaaaacgttcttcggggcgaaaactctcaaggatcttaccgctgttgagatccagttcgatgtaacccactcgtgcacccaactgatc
ttcagcatcttttactttcaccagcgtttctgggtgagcaaaaacaggaaggcaaaatgccgcaaaaaagggaataagggcgacacggaaa
tgttgaatactcatactcttcctttttcaatattattgaagcatttatcagggttattgtctcatgagcggatacatatttgaatgtatttagaaaaataaa
caaataggggttccgcgcacatttccccgaaaagtgccacctgacgcgccctgtagcggcgcattaagcgcggcgggtgtggtggttacgc
gcagcgtgaccgctacacttgccagcgccctagcgcccgctcctttcgctttcttcccttcctttctcgccacgttcgccggctttccccgtcaagct
ctaaatcgggggctccctttagggttccgatttagtgctttacggcacctcgaccccaaaaaacttgattagggtgatggttcacgtagtgggcca
tcgccctgatagacggtttttcgccctttgacgttggagtccacgttctttaatagtggactcttgttccaaactggaacaacactcaaccctatctcg
gtctattcttttgatttataagggattttgccgatttcggcctattggttaaaaaatgagctgatttaacaaaaatttaacgcgaattttaacaaaatatt
aacgcttacaatttgccattcgccattcaggctgcgcaactgttgggaagggcgatcggtgcgggcctcttcgctattacgccagcccaagcta
ccatgataagtaagtaatattaaggtacgggaggtacttggagcggccgcaataaaatatctttattttcattacatctgtgtgttggttttttgtgtga
atcgatagtactaacatacgctctccatcaaaacaaaacgaaacaaaacaaactagcaaaataggctgtccccagtgcaagtgcaggtgc
cagaacatttctctatcgataggtaccgattagtgaacggatctcgacggtatcgatcacgagactagcctcgagcggccgccccccttcacc

Sth1a-gRNA vector sequence (U6 promoter-gRNA scaffold-EGFP-ori-AmpR-AmpR promoter)

gagggcctatttcccatgattccttcatatttgcatatacgatacaaggctgttagagagataattggaattaatttgactgtaaacacaaagatatt
agtacaaaatacgtgacgtagaaagtaataatttcttgggtagtttgcagttttaaaattatgttttaaaatggactatcatatgcttaccgtaacttg
aaagtatttcgatttcttggctttatatatcttgtggaaaggacgaaacaccgtgagaccgagagagggtctcaGTTTTTGTACTCTCA
GAAATGCAGAAGCTACAAAGATAAGGCTTCATGCCGAAATCAACACCCTGTCATTTTATGGCAGG
GTGTTTTtttttttaaagaattctcgacctcgagacaaatggcagtattcatccacaattttaaaagaaaaggggggattggggggtacagt
gcaggggaaagaatagtagacataatagcaacagacatacaaactaaagaattacaaaaacaaattacaaaaattcaaaattttcgggttt
attacagggacagcagagatccactttggccgcggctcgagggggttggggttgcgccttttccaaggcagccctgggtttgcgcagggacg
cggctgctctgggcgtggttccgggaaacgcagcggcgccgaccctgggactcgcacattcttcacgtccgttcgcagcgtcacccggatctt
cgccgctacccttgtgggccccccggcgacgcttcctgctccgcccctaagtcgggaaggttccttgcggttcgcggcgtgccggacgtgaca
aacggaagccgcacgtctcactagtaccctcgcagacggacagcgccagggagcaatggcagcgcgccgaccgcgatgggctgtggcc
aatagcggctgctcagcagggcgcgccgagagcagcggccgggaaggggcggtgcgggaggcggggtgtggggcggtagtgtgggcc
ctgttcctgcccgcgcggtgttccgcattctgcaagcctccggagcgcacgtcggcagtcggctccctcgttgaccgaatcaccgacctctctcc
ccaggggggatccatggtgagcaagggcgaggagctgttcaccggggtggtgcccatcctggtcgagctggacggcgacgtaaacggcca
caagttcagcgtgtccggcgagggcgagggcgatgccacctacggcaagctgaccctgaagttcatctgcaccaccggcaagctgcccgt
gccctggcccaccctcgtgaccaccctgacctacggcgtgcagtgcttcagccgctaccccgaccacatgaagcagcacgacttcttcaagt
ccgccatgcccgaaggctacgtccaggagcgcaccatcttcttcaaggacgacggcaactacaagacccgcgccgaggtgaagttcgag
ggcgacaccctggtgaaccgcatcgagctgaagggcatcgacttcaaggaggacggcaacatcctggggcacaagctggagtacaacta
caacagccacaacgtctatatcatggccgacaagcagaagaacggcatcaaggtgaacttcaagatccgccacaacatcgaggacggc
agcgtgcagctcgccgaccactaccagcagaacacccccatcggcgacggccccgtgctgctgcccgacaaccactacctgagcaccca
gtccgccctgagcaaagaccccaacgagaagcgcgatcacatggtcctgctggagttcgtgaccgccgccgggatcactctcggcatgga
cgagctgtacaagtaaagcggccgcgactctagatcataatcagccataccacatttgtagaggttttacttgctttaaaaaacctcccacacct
ccccctgaacctgaaacataaaatgaatgcaattgttgttgttaacttgtttattgcagcttataatggttacaaataaagcaatagcatcacaaat
ttcacaaataaagcatttttttcactgcattctagttgtggtttgtccaaactcatcaatgtatcttagtcgaccgatgcccttgagagccttcaaccca
gtcagctccttccggtgggcgcggggcatgactatcgtcgccgcacttatgactgtcttctttatcatgcaactcgtaggacaggtgccggcagc
gctcttccgcttcctcgctcactgactcgctgcgctcggtcgttcggctgcggcgagcggtatcagctcactcaaaggcggtaatacggttatcc
acagaatcaggggataacgcaggaaagaacatgtgagcaaaaggccagcaaaaggccaggaaccgtaaaaaggccgcgttgctggc
gtttttccataggctccgcccccctgacgagcatcacaaaaatcgacgctcaagtcagaggtggcgaaacccgacaggactataaagatac
caggcgtttccccctggaagctccctcgtgcgctctcctgttccgaccctgccgcttaccggatacctgtccgcctttctcccttcgggaagcgtgg

cgctttctcatagctcacgctgtaggtatctcagttcggtgtaggtcgttcgctccaagctgggctgtgtgcacgaaccccccgttcagcccgacc
gctgcgccttatccggtaactatcgtcttgagtccaacccggtaagacacgacttatcgccactggcagcagccactggtaacaggattagca
gagcgaggtatgtaggcggtgctacagagttcttgaagtggtggcctaactacggctacactagaagaacagtatttggtatctgcgctctgctg
aagccagttaccttcggaaaaagagttggtagctcttgatccggcaaacaaaccaccgctggtagcggtggtttttttgtttgcaagcagcagat
tacgcgcagaaaaaaaggatctcaagaagatcctttgatcttttctacggggtctgacgctcagtggaacgaaaactcacgttaagggattttg
gtcatgagattatcaaaaaggatcttcacctagatccttttaaattaaaaatgaagttttaaatcaatctaaagtatatatgagtaaacttggtctga
cagttaccaatgcttaatcagtgaggcacctatctcagcgatctgtctatttcgttcatccatagttgcctgactccccgtcgtgtagataactacga
tacgggagggcttaccatctggccccagtgctgcaatgataccgcgggacccacgctcaccggctccagatttatcagcaataaaccagcc
agccggaagggccgagcgcagaagtggtcctgcaactttatccgcctccatccagtctattaattgttgccgggaagctagagtaagtagttcg
ccagttaatagtttgcgcaacgttgttgccattgctacaggcatcgtggtgtcacgctcgtcgtttggtatggcttcattcagctccggttcccaacg
atcaaggcgagttacatgatcccccatgttgtgcaaaaaagcggttagctccttcggtcctccgatcgttgtcagaagtaagttggccgcagtgtt
atcactcatggttatggcagcactgcataattctcttactgtcatgccatccgtaagatgcttttctgtgactggtgagtactcaaccaagtcattctg
agaatagtgtatgcggcgaccgagttgctcttgcccggcgtcaatacgggataataccgcgccacatagcagaactttaaaagtgctcatcatt
ggaaaacgttcttcggggcgaaaactctcaaggatcttaccgctgttgagatccagttcgatgtaacccactcgtgcacccaactgatcttcag
catcttttactttcaccagcgtttctgggtgagcaaaaacaggaaggcaaaatgccgcaaaaaagggaataagggcgacacggaaatgttg
aatactcatactcttcctttttcaatattattgaagcatttatcagggttattgtctcatgagcggatacatatttgaatgtatttagaaaaataaacaaa
taggggttccgcgcacatttccccgaaaagtgccacctgacgcgccctgtagcggcgcattaagcgcggcgggtgtggtggttacgcgcagc
gtgaccgctacacttgccagcgccctagcgcccgctcctttcgctttcttcccttcctttctcgccacgttcgccggctttccccgtcaagctctaaat
cgggggctccctttagggttccgatttagtgctttacggcacctcgaccccaaaaaacttgattagggtgatggttcacgtagtgggccatcgcc
ctgatagacggtttttcgccctttgacgttggagtccacgttctttaatagtggactcttgttccaaactggaacaacactcaaccctatctcggtcta
ttcttttgatttataagggattttgccgatttcggcctattggttaaaaaatgagctgatttaacaaaaatttaacgcgaattttaacaaaatattaacg
cttacaatttgccattcgccattcaggctgcgcaactgttgggaagggcgatcggtgcgggcctcttcgctattacgccagcccaagctaccatg
ataagtaagtaatattaaggtacgggaggtacttggagcggccgcaataaaatatctttattttcattacatctgtgtgttggttttttgtgtgaatcga
tagtactaacatacgctctccatcaaaacaaaacgaaacaaaacaaactagcaaaataggctgtccccagtgcaagtgcaggtgccagaa
catttctctatcgataggtaccgattagtgaacggatctcgacggtatcgatcacgagactagcctcgagcggccgccccccttcacc

TadA/TadA*(TadA WT-32aa linker- TadA*)

tctgaagtcgagtttagccacgagtattggatgaggcacgcactgaccctggcaaagcgagcatgggatgaaagagaagtccccgtgggc
gccgtgctggtgcacaacaatagagtgatcggagagggatggaacaggccaatcggccgccacgaccctaccgcacacgcagagatca
tggcactgaggcagggaggcctggtcatgcagaattaccgcctgatcgatgccaccctgtatgtgacactggagccatgcgtgatgtgcgca
ggagcaatgatccacagcaggatcggaagagtggtgttcggagcacgggacgccaagaccggcgcagcaggctccctgatggatgtgct
gcaccaccccggcatgaaccaccgggtggagatcacagagggaatcctggcagacgagtgcgccgccctgctgagcgatttctttagaat
gcggagacaggagatcaaggcccagaagaaggcacagagctccaccgactctggaggatctagcggaggatcctctggaagcgagac
accaggcacaagcgagtccgccacaccagagagctccggcggctcctccggaggatcctctgaggtggagttttcccacgagtactggatg
agacatgccctgaccctggccaagagggcacgcgatgagagggaggtgcctgtgggagccgtgctggtgctgaacaatagagtgatcgg
cgagggctggaacagagccatcggcctgcacgacccaacagcccatgccgaaattatggccctgagacagggcggcctggtcatgcaga
actacagactgattgacgccaccctgtacgtgacattcgagccttgcgtgatgtgcgccggcgccatgatccactctaggatcggccgcgtggt
gtttggcgtgaggaacgcaaaaaccggcgccgcaggctccctgatggacgtgctgcactaccccggcatgaatcaccgcgtcgaaattacc
gagggaatcctggcagatgaatgtgccgccctgctgtgctatttctttcggatgcctagacaggtgttcaatgctcagaagaaggcccagagct
ccaccgac

Human APOBEC3A (Y130F)

gaagccagcccagcatccgggcccagacacttgatggatccacacatattcacttccaactttaacaatggcattggaaggcataagaccta
cctgtgctacgaagtggagcgcctggacaatggcacctcggtcaagatggaccagcacaggggctttctacacaaccaggctaagaatctt
ctctgtggcttttacggccgccatgcggagctgcgcttcttggacctggttccttctttgcagttggacccggcccagatctacagggtcacttggtt
catctcctggagcccctgcttctcctggggctgtgccggggaagtgcgtgcgttccttcaggagaacacacacgtgagactgcgtatcttcgctg

cccgcatctTtgattacgacccccctatataaggaggcactgcaaatgctgcgggatgctggggcccaagtctccatcatgacctacgatgaat
ttaagcactgctgggacacctttgtggaccaccagggatgtcccttccagccctgggatggactagatgagcacagccaagccctgagtggg
aggctgcgggccattctccagaatcagggaaac

Supplementary Table 3. The oligo sequences used for constructing gRNAs in the present study and the oligos synthesized for constructing gRNAs and primers used for detecting targeting efficiencies in blastocysts or mice.

Oligo	F (BsaI cohesive ends - sgRNA oligo)	R (BsaI cohesive ends- sgRNA oligo)
Sgo-6	ACCGcggacctctacctctaactcacaa	AAACttgtgagttagaggtagaggtccg
Sgo-7	ACCGgcttcgccaggaatatatagcttc	AAACgaagctatatattcctggcgaagc
Sgo-8	ACCGcaggaaccacttgaagcgtgcagt	AAACactgcacgcttcaagtggttcctg
Sgo-9	ACCGagagagccacatctacttgcaact	AAACagttgcaagtagatgtggctctct
Sgo-10	ACCGaagagatcctcctgccttggcctc	AAACgaggccaaggcaggaggatctctt
Sgo-11	ACCGgaagaagttccagaccaaggaagt	AAACacttccttggtctggaacttcttc
Sgo-12	ACCGagggcctccaacattagacacaca	AAACtgtgtgtctaatgttggaggccct
Sgo-13	ACCGcgtggacacttccagctggatttg	AAACcaaatccagctggaagtgtccacg
Sgo-14	ACCGacttgtaatcctaacctttgggac	AAACgtcccaaaggttaggattacaagt
Sgo-16	ACCGgcacacgcctaatcacaaatacca	AAACtggtatttgtgattaggcgtgtgc
Sgo-17	ACCGtgtatgcaacttccaggtttgtct	AAACagacaaacctggaagttgcataca
Sgo-19	ACCGtgtcaagtcctcaggttccaccat	AAACatggtggaacctgaggacttgaca
Sgo-20	ACCGaacacagagacaccggccactggt	AAACaccagtggccggtgtctctgtgtt
Sgo-21	ACCGaggcataagagcacctgctattaga	AAACctaatagcaggtgctcttatgcct
Sgo-22	ACCGtgctgcctccaacaagacaagcca	AAACtggcttgtcttgttggaggcagca
Sgo-23	ACCGgggaggatccaaaccacaacggaa	AAACttccgttgtggtttggatcctccc
Sgo-24	ACCGctgagaagccaggtcctaggccaa	AAACttggcctaggacctggcttctcag
Sgo-25	ACCGatgagcctggtccatggagagttt	AAACaaactctccatggaccaggctcat
Sth1a-3	ACCGctttgcgaccagttaactga	AAACtcagttaactggtcgcaaag
Sth1a-6	ACCGagcatcagccgtataagtca	AAACtgacttatacggctgatgct
Sth1a-9	ACCGcgagacaagcctagccaaca	AAACtgttggctaggcttgtctcg
Sth1a-11	ACCGtacagcgcggttccagggct	AAACagccctggaaccgcgctgta
Sth1a-13	ACCGgtggacacttccagctggat	AAACatccagctggaagtgtccac
Sth1a-18	ACCGgagtgccttacttgggagct	AAACagctcccaagtaaggcactc
Sth1a-19	ACCGcacatgccactgcacacagc	AAACgctgtgtgcagtggcatgtg
Sth1a-20	ACCGaggctgtagccagtctggag	AAACctccagactggctacagcct
Sth1a-25	ACCGccttgcagctctaagagact	AAACagtctcttagagctgcaagg
sgAr-1	ACCGggaactcgatcgcatcattgcatg	AAACcatgcaatgatgcgatcgagttcc
sgAr-2	ACCGccatcttgtcgtctccggaa	AAACttccggagacgacaagatgg
sgAr-1 primer	GTTCCTTCATGGGCATGCTGC	CACTGGGCTCTATCAAGCTGC
sgAr-2 primer	GGAAAGAAGCTCCGGAGACA	GGAGAAGCTAGATCTAATGCAATG

Supplementary Table 4. The primer sequences for constructing ancSgo-BE4, ancSth1a-BE4, ancSgo-ABEmax, ancSth1a-ABEmax, Sgo-A3A-BE4, Sth1a-A3A-BE4, and their gRNA expression vectors.

		Primer sequence
ABE	P-Sgo-FOR	ggcagcagcggggggtcaAACGGCCTGGTGCTGGGCCTGG
	P-Sgo-REV	tctttttgagccgccagaCTTCTTGAACTTGAGCTTGGGT
	Sgo-backbone-FOR	tctggcggctcaaaaagaaccgccgacggcagcgaa
	Sgo-backbone-REV	tgacccccgctgctgcccccgctgctttcaggtgt
	P-Sth1A-FOR	ggcagcagcggggggtcaAGCGACCTGGTTCTGGGCCTAG
	P-Sth1A-REV	tctttttgagccgccagaGAAATCCAACTTAGGCTTGTCG
	Sth1a-backbone-FOR	tctggcggctcaaaaagaaccgccgacggcagcgaa
	Sth1a-backbone-REV	tgacccccgctgctgcccccgctgctttcaggtgt
A3A	Sgo-A3A-FOR	ccaaagaagaagcggaaagtcgaagccagcccagcatccgg
	Sgo-A3A-REV	cctccgctagatcctccagagtttccctgattctggagaatg
	Sgo-Backbone-FOR	tctggaggatctagcggaggatcctctggcagcgagacac
	Sgo-Backbone-REV	gactttccgcttcttctttggtgactcgaactcgcttccg
	Sth1a-A3A-FOR	ccaaagaagaagcggaaagtcgaagccagcccagcatccgg
	Sth1a-A3A-REV	cctccgctagatcctccagagtttccctgattctggagaatg
	Sth1a-Backbone-FOR	tctggaggatctagcggaggatcctctggcagcgagacaccag
	Sth1a-Backbone-REV	gactttccgcttcttctttggtgactcgaactcgcttccg

Supplementary Table 5. The primers used for PCR amplification.

	FOR	REV
Sgo6	TGGAGGACCCGTGAGATAC	CTATCAGTTGGGATTTCTCAGG
Sgo7	GACACAGCCAGTGTAA	TCCATGTCAAAGAGAC
Sgo8	GGTTGAAAGGAGGTTAACATCT	CATTCTGGTACCATGACAGC
Sgo9	GATGCGCTATCATTCATTGT	CAATCAAGTGTCCAAGGCATG
Sgo10	CTCTGTTTGCTATCCCATAGTG	GTTCAAAGCTCTGGCTATTTC
Sgo11	GAAGTTACTTAACCTTAGAGCC	TCTAGCAAAGCCATTCAGAAC
Sgo12	AAGGCTGCATGGTTGGTC	ACGCACACCTCACTTGAAC
Sgo13	TTCCGTGCACAAGGATCAG	GAACCAAATATCCATCCCTCC
Sgo14	CCATGCCCAGATGACTTTCTTAG	GAGAATCACTTGAACCCTGAAAG
Sgo16	GTGTCTGTTTATGGTTGACAAG	GGTGAAACTGCTTCCTGTG
Sgo17	ATATGGTGGCACCACGATG	GACCACATATCCCAGACTCC
Sgo19	CCATGGCCGTTAACAGTTAG	GCTCCAGGGATATGACTCTG
Sgo20	TCAAGTGCTTCTTTACTGGC	CAGAGTAAAGCCAGGAAGATC
Sgo21	ATAGATCCGAAAGATGTGCTC	TGCTATTTGATGAAAGCCAG
Sgo22	CCTGTATACCTTTGAGCAGG	TGCAGAACTCAAATGAGACAC
Sgo23	CATTTGACTATGTTCGGGTGC	TTAGGTCAGGGAAACTCGC
Sgo24	GCGAGTTTCCCTGACCTAA	CAACAACCAGGCTTTCGTG
Sgo25	CAGATGGATGCCTGTGTC	TTCACCCTGACTCCTAGTAG
Sth1a3	GCCGCAAGACTAGTGCAAG	AAGTCCATGGCGCTCTTTC
Sth1a6	GGCCTACCAAAAGTCATTGAC	GATGTTAACCTCCTTTCAACC
Sth1a9	GACTCTTGAAGGAGCCTACTC	GGGTTCGAGCAATTCTCCTG
Sth1a11	CAGTTACAGCTAGCATCCAG	TGAAAGACTTGTGTGTTTGG
Sth1a13	CCGTGCACAAGGATCAGTG	TGAACCAAATATCCATCCCTC
Sth1a18	CTGCAATCCAGCCTGGATG	GCAGCCTGGGCAAGTAGAAC
Sth1a19	GAGCTGACCAGCCTGTGTC	GATCATCTCTGCTATGGTAGC
Sth1a20	CAGTGGATCGACCAGCTTG	ACATCTCTAGGTTAGAACCCTG
Sth1a25	GTCTTGTCCCACATTTGGAC	GTCCATCTGTGTGTCTATGTC

Supplementary Table 6. The primers used to amplify potential off-targeting sites.

Sgo-11-OT-1	CCTTCAACATGGTTCTGTCTG	TAAGGAGGATGGTAGTCATCC
Sgo-11-OT-2	GGCTAGCCAGCAGTTAGGAC	CACCGTCCTGACTCATTTCC
Sgo-11-OT-3	CTGCCAGGCACTGTGTAAG	GTCCGGTAGGTAACTGTTTAATG
Sgo-11-OT-4	GTTGACTACTTGCCATGTGC	GTAAGACAGTGTAATACAGTGGAC
Sgo-11-OT-5	GCTCACAACAATTCTGTGAC	CCTTTGGTTGATATGTGCAAG
Sgo-11-OT-6	ACAACCACTCCCTTTCAGG	GTAGACTGTCAAATCTCAGAGC
Sgo-11-OT-7	GAAGGCTAGCCAGCAGTTAG	CGTCCTGACTCATTTCCAG
Sgo-11-OT-8	CCTGCAGAGATTCACAGAAGC	AGTAAGCGTTAGCCAACTC
Sgo-11-OT-9	GGATGTCAGATAGAGTTGGATG	TCTGCACAATGGCCTTCAAG
Sgo-11-OT-10	CTAGCATCCACTAAACCTGG	CGGTTTGTTATCAATACAGG
Sgo-11-OT-11	GCCAGGCACTGTGTAAGTC	CTTGTCCGGTAGGTAACTG
Sgo-14-OT-1	GAAGAATGAAGTTGCTGATAGC	CCATTGCTAGAACAGTGTCTG
Sgo-14-OT-2	GCCTGTGATTCACAATCCTAAG	GTGCACCACCATATTCAGC
Sgo-14-OT-3	GATGCAAATAACCGTGGATG	GTTAAGACAATGCTGCATGTG
Sgo-14-OT-4	GACTTTCTATTTCAACCAAGGGAG	CTCAGCCTCTTGAGCAGTTG
Sgo-14-OT-5	GACTGAGTGAGTGAATGAACAG	GACACAGTGCCTTTAGATTAAGG
Sgo-23-OT-1	GGCAATAGGGTCATTACAGATG	AAGTCAAGGTCTTGGCAGG
Sgo-23-OT-2	GAGTCCCTGCACACTCATAG	GCTGTCTACATCTACAGGATG
Sgo-23-OT-3	GTGCATACAAATATGTGCTCAG	CCTACAATACATGCATGCATATC
Sgo-23-OT-4	GTGAAGCTACTGAGCTAAGGACTAG	CGTTGTGGCCTTATGGCAAAG
Sgo-23-OT-5	CAAAGAGGGTTATTCACTAGTCC	AAGGCAATTTGTGCAGTGC
Sth1a-3-OT-1	CAAGCTCTCCAGGTGATTCTG	GGCCTCACCTCATATTCATAAC
Sth1a-3-OT-2	GGAGCTGGATTGATCCTTCAG	GGTGGCCATGTGCAACAC
Sth1a-3-OT-3	CACTAAGCCTCAGTTGTCCTG	CAGTGAGCCATGATCGTGC
Sth1a-3-OT-4	GGCAACAGAGTGAGAACCTG	CCCTCATTGACTCAAGTAGGTAC
Sth1a-3-OT-5	GCTAAGGGCCATAGTCAGG	CATCCATCCACCCAGACAATC
Sth1a-3-OT-6	CAGTATGCATGGCTCAGAGTC	GAACTCCCGTGTTCTTCCTTG
Sth1a-3-OT-7	GACTGAGCCAGCGCTACTG	GAAGACAACCTGCCTCCAAT
Sth1a-3-OT-8	CATGAGCCACCACACCTG	GCCAAGCACCAGCACTG
Sth1a-3-OT-9	ACCCAGGCAGAGTTGGTT	AGTGTCCCAGTCAGTGCTC
Sth1a-3-OT-10	GGGTAGACCTTGTAAGACAAGG	GCATGTGTTCCTCCCAGGAA
Sth1a-3-OT-11	GCTTCTTGGAACATTCATTTCCTG	GCCAGGCATTGTTGAAGTG
Sth1a-3-OT-12	CCTAAGAATACATACCACTTGCTTC	CTCCTGTGTTAAACAGAAACTCATGG
Sth1a-3-OT-13	CCTACCATGAACTTCCTCTGC	CCCTCAACATGACATTGAGAGTC
Sth1a-11-OT-1	GTCCTGCATCCCAGGAAC	GGTGGAGAGTGCACTGCA
Sth1a-11-OT-2	GGACTTCCAGCCTCTAGAAC	CTTCCCTGCTTGCCAAGAC
Sth1a-11-OT-3	GGAGCCTGAAGCATGGC	AGCATCGTGGTGGTGCA
Sth1a-11-OT-4	TCCTGACTCTGGTGAGGTT	GCATTCCCCAAATCACTTCTC
Sth1a-11-OT-5	CAGGCTGGTCTTGAGCTC	GGCAGCACCAAGGACAAG
Sth1a-11-OT-6	GTTGTAGTAGCTAGTGTTTCCCT	CCGGCTATCAGATAGTTGTGT

Sth1a-11-OT-7	CCCACAAGTGTCTCAGAGC	GGACTGTATTACAGCACCAGG
Sth1a-11-OT-8	GCGGAATCAAAGAAGGCAGA	GGATCCTAATTAGGTACTCTTATGTCATC
Sth1a-11-OT-9	GCCATTGCACTCAGCCTG	CACCCAACGGTCTTCCTG
Sth1a-11-OT-10	CCCTGGGAATGATAACTCACG	GCCTCTTCGTACACTACTTAGG
Sth1a-11-OT-11	CGCTCAGAATCCCAGACAG	GCAGTTCACAACCTGTAGCA
Sth1a-11-OT-12	GGGAGATTTGGAAGATGGAAGTG	CCCTGGAAACTGTTAACTCCA
Sth1a-11-OT-13	GGATCAGCTGCATCTGCAG	CCACCGTCAGAATTGGAGG
Sth1a-11-OT-14	CCCATTGCTGTGTCCTTGAC	GGAGAGAAATCATGCTTGCAG
Sth1a-18-OT-1	TGGTTGGCCTCGTGGATG	CCACTCATCAGCACGTAGAC
Sth1a-18-OT-2	GGTGTCCACTGTCATCTGTG	TCCACACGGAACTGCCTC
Sth1a-18-OT-3	TCCAAACTAAGCACTTACCGT	GCAACTGTTCCTACTGAAAGGC
Sth1a-18-OT-4	GAGTGCAGTGTCTGTAAGGTG	GAGTTGGCTTGGCTGAGAC
Sth1a-18-OT-5	CCCAAATAGTAAGTCTGGACTCAG	GGAGCTCCTAGAATGTTGCTG
Sth1a-18-OT-6	CCCAAGCCAGCAAACACTAAC	GGCCTTCACTAAAGTATCTGGAC
Sth1a-18-OT-7	AGCTCCCTAGGTGATTCTAGTG	GCTACCAGGCTTCTTATGAAGC
Sth1a-18-OT-8	GGAATCAGCTCCACAGCATC	CCGAGGTGTGACTGATCTAG
Sth1a-18-OT-9	GGACCCAAGAGAGGTGCA	CTCCCTGTCCAATTCTGTGC
Sth1a-18-OT-10	GGCCTTACCTGATCTTCTCTG	CCTACAATGGATTGGGCTACC
Sth1a-18-OT-11	CCAAGTACCATGCTTAGCACG	GCTTGTGTGACCATGCATTG
Sth1a-18-OT-12	GGGTGATCCCATGTGGAG	GAACTCTGAGCACTTCTCCGT
Sth1a-18-OT-13	GGAAGGGAGTCACTCTTGATC	CCAATCCTGGCTGCTCTTC
Sth1a-18-OT-14	GCATCTATGCCAAGCCACA	GAGGTTGCAGTGAGCCAAG
Sth1a-18-OT-15	GCCACATGGAACTGTGAGTC	CCTCCTTGGACCTGAGCT
Sth1a-18-OT-16	GCTGCTGCTGTGGATGC	AGGTAGTAGCGAGTCACCTC
Sth1a-18-OT-17	CCCAAAGCACCCAATCCT	GCGACATAAAGGCCTCACC
Sth1a-18-OT-18	CTCCCTCCTTTATCATGCCAT	GGAGAAGCAGGAAGTGTTAACATC
Sth1a-18-OT-19	CAGGGATCTGCAGAGAGGAC	GGGCTCATGAGCTTTGTCATC
Sth1a-18-OT-20	CCCATACTTAAGCCACACACA	AGAGCACCAGGAGGAAGAG
Ar-Sth1a-OT-1	CCACGCTTAGGACCACTGTG	GCTCAGGAGCCATATTAGCTGG
Ar-Sth1a-OT-2	CAGTGGCCATGGATCCTGT	CCATTGCTTCCATCAGCAGC
Ar-Sth1a-OT-3	CCGTCACCTTAGCAGGCTC	GGCTGGAGGATCCTGTGTCC
Ar-Sth1a-OT-4	GTTGGCTCCACCCTTCTGG	CGGTAATGTTCACGTGGTTCTG
Ar-Sth1a-OT-5	CTTACGAGATGGTCTCCAGTTCTC	GGAGGAGCCACCCTAACAGTAG
Ar-Sth1a-OT-6	GGCACCCATATGCCAAAGTACAC	CTTGTGAAGCTATGACCAGCAG
Ar-Sth1a-OT-7	GGAGTCCTCAGCCAACCTATTC	CCCACCAAACACAACGCGA
Ar-Sth1a-OT-8	CCGTGTGACTTCAGGATGCTC	CTGGCAAGGGTGCTGTGAAC
Ar-Sth1a-OT-9	CGGAGCCCTTCACAAGATTGAG	GTGGCAGGTGTCAGAAGACTAG
Ar-Sth1a-OT-10	GCACCAGCATAGCCTCACAAG	GTTGGCAGATAGTGAGTACAGGC
Ar-Sth1a-OT-11	CCTGAAGCTGCTGTGTACATCC	CAGCAAACATTTACTGAGCACTGG
Ar-Sth1a-OT-12	GAGATCAACCCTGAGTTCTGCCTTG	GCTAACCTCCCAATCTGCTTGG

Supplementary Table 7. The synthesized oligos for PAM and gRNA mismatch test and primers used for PCR amplification.

	FOR	REV
Sgo-14	aattacttgtaatcctaacctttgggacACAAAGgtac	CTTTGTgtcccaaaggttaggattacaagt
PAM-Sgo-14-1	aattacttgtaatcctaacctttgggacACTAAGgtac	CTTAGTgtcccaaaggttaggattacaagt
PAM-Sgo-14-2	aattacttgtaatcctaacctttgggacACCAAGgtac	CTTGGTgtcccaaaggttaggattacaagt
PAM-Sgo-14-3	aattacttgtaatcctaacctttgggacACGAAGgtac	CTTCGTgtcccaaaggttaggattacaagt
PAM-Sgo-14-4	aattacttgtaatcctaacctttgggacACATAGgtac	CTATGTgtcccaaaggttaggattacaagt
PAM-Sgo-14-5	aattacttgtaatcctaacctttgggacACACAGgtac	CTGTGTgtcccaaaggttaggattacaagt
PAM-Sgo-14-6	aattacttgtaatcctaacctttgggacACAGAGgtac	CTCTGTgtcccaaaggttaggattacaagt
PAM-Sgo-14-7	aattacttgtaatcctaacctttgggacACAATGgtac	CATTGTgtcccaaaggttaggattacaagt
PAM-Sgo-14-8	aattacttgtaatcctaacctttgggacACAACGgtac	CGTTGTgtcccaaaggttaggattacaagt
PAM-Sgo-14-9	aattacttgtaatcctaacctttgggacACAAGGgtac	CCTTGTgtcccaaaggttaggattacaagt
PAM-Sgo-14-10	aattacttgtaatcctaacctttgggacACAAAAgtac	TTTTGTgtcccaaaggttaggattacaagt
PAM-Sgo-14-11	aattacttgtaatcctaacctttgggacACAAATgtac	ATTTGTgtcccaaaggttaggattacaagt
PAM-Sgo-14-12	aattacttgtaatcctaacctttgggacACAAACgtac	GTTTGTgtcccaaaggttaggattacaagt
Sgo-23	aattgggaggatccaaaccacaacggaaCAAAAGgtac	CTTTTGttccgttgtggtttggatcctccc
PAM-Sgo-23-1	aattgggaggatccaaaccacaacggaaCATAAGgtac	CTTATGttccgttgtggtttggatcctccc
PAM-Sgo-23-2	aattgggaggatccaaaccacaacggaaCACAAGgtac	CTTGTGttccgttgtggtttggatcctccc
PAM-Sgo-23-3	aattgggaggatccaaaccacaacggaaCAGAAGgtac	CTTCTGttccgttgtggtttggatcctccc
PAM-Sgo-23-4	aattgggaggatccaaaccacaacggaaCAATAGgtac	CTATTGttccgttgtggtttggatcctccc
PAM-Sgo-23-5	aattgggaggatccaaaccacaacggaaCAACAGgtac	CTGTTGttccgttgtggtttggatcctccc
PAM-Sgo-23-6	aattgggaggatccaaaccacaacggaaCAAGAGgtac	CTCTTGttccgttgtggtttggatcctccc
PAM-Sgo-23-7	aattgggaggatccaaaccacaacggaaCAAATGgtac	CATTTGttccgttgtggtttggatcctccc
PAM-Sgo-23-8	aattgggaggatccaaaccacaacggaaCAAACGgtac	CGTTTGttccgttgtggtttggatcctccc
PAM-Sgo-23-9	aattgggaggatccaaaccacaacggaaCAAAGGgtac	CCTTTGttccgttgtggtttggatcctccc
PAM-Sgo-23-10	aattgggaggatccaaaccacaacggaaCAAAAAgtac	TTTTTGttccgttgtggtttggatcctccc
PAM-Sgo-23-11	aattgggaggatccaaaccacaacggaaCAAAATgtac	ATTTTGttccgttgtggtttggatcctccc
PAM-Sgo-23-12	aattgggaggatccaaaccacaacggaaCAAAACgtac	GTTTTGttccgttgtggtttggatcctccc
Sth-18	aattgagtgccttacttgggagctTAGTAAAgtac	TTTACTAagctcccaagtaaggcactc
PAM- Sth-18-1	aattgagtgccttacttgggagctTGGTAAAgtac	TTTACCAagctcccaagtaaggcactc
PAM-Sth-18-2	aattgagtgccttacttgggagctTAATAAAgtac	TTTATTAagctcccaagtaaggcactc
PAM- Sth-18-3	aattgagtgccttacttgggagctTACTAAAgtac	TTTAGTAagctcccaagtaaggcactc
PAM- Sth-18-4	aattgagtgccttacttgggagctTATTAAAgtac	TTTAATAagctcccaagtaaggcactc
PAM- Sth-18-5	aattgagtgccttacttgggagctTAGGAAAgtac	TTTCCTAagctcccaagtaaggcactc
PAM- Sth-18-6	aattgagtgccttacttgggagctTAGTCAAgtac	TTGACTAagctcccaagtaaggcactc
PAM- Sth-18-7	aattgagtgccttacttgggagctTAGTAGAgtac	TCTACTAagctcccaagtaaggcactc
Sth-25	aattccttgcagctctaagagactCTGCGAAgtac	TTCGCAGagtctcttagagctgcaagg
PAM- Sth-25-1	aattccttgcagctctaagagactCGGCGAAgtac	TTCGCCGagtctcttagagctgcaagg
PAM- Sth-25-2	aattccttgcagctctaagagactCTACGAAgtac	TTCGTAGagtctcttagagctgcaagg
PAM- Sth-25-3	aattccttgcagctctaagagactCTCCGAAgtac	TTCGGAGagtctcttagagctgcaagg
PAM- Sth-25-4	aattccttgcagctctaagagactCTTCGAAgtac	TTCGAAGagtctcttagagctgcaagg

PAM- Sth-25-5	aattccttgcagctctaagagactCTGGGAAgtac	TTCCCAGagtctcttagagctgcaagg
PAM- Sth-25-6	aattccttgcagctctaagagactCTGCCAAgtac	TTGGCAGagtctcttagagctgcaagg
PAM- Sth-25-7	aattccttgcagctctaagagactCTGCGGAgtac	TCCGCAGagtctcttagagctgcaagg
Sgo-14	accgacttgtaatcctaacctttgggac	aaacgtcccaaaggttaggattacaagt
gRNA-Sgo-14-1	accgGTttgtaatcctaacctttgggac	aaacgtcccaaaggttaggattacaaAC
gRNA-Sgo-14-2	accgacCCgtaatcctaacctttgggac	aaacgtcccaaaggttaggattacGGgt
gRNA-Sgo-14-3	accgacttACaatcctaacctttgggac	aaacgtcccaaaggttaggattGTaagt
gRNA-Sgo-14-4	accgacttgtGGtcctaacctttgggac	aaacgtcccaaaggttaggaCCacaagt
gRNA-Sgo-14-5	accgacttgtaaCTctaacctttgggac	aaacgtcccaaaggttagAGttacaagt
gRNA-Sgo-14-6	accgacttgtaatcTCaacctttgggac	aaacgtcccaaaggttGAgattacaagt
gRNA-Sgo-14-7	accgacttgtaatcctGGcctttgggac	aaacgtcccaaaggCCaggattacaagt
gRNA-Sgo-14-8	accgacttgtaatcctaaTTtttgggac	aaacgtcccaaaAAttaggattacaagt
gRNA-Sgo-14-9	accgacttgtaatcctaaccCCtgggac	aaacgtcccaGGggttaggattacaagt
gRNA-Sgo-14-10	accgacttgtaatcctaaccttCAggac	aaacgtccTGaaggttaggattacaagt
gRNA-Sgo-14-11	accgacttgtaatcctaacctttgAAac	aaacgtTTcaaaggttaggattacaagt
gRNA-Sgo-14-12	accgacttgtaatcctaacctttgggGT	aaacACcccaaaggttaggattacaagt
Sth-6	accgagcatcagccgtataagtca	aaactgacttatacggctgatgct
gRNA-Sth-6-1	accgGAcatcagccgtataagtca	aaactgacttatacggctgatgTC
gRNA-Sth-6-2	accgagTGtcagccgtataagtca	aaactgacttatacggctgaCAct
gRNA-Sth-6-3	accgagcaCTagccgtataagtca	aaactgacttatacggctAGtgct
gRNA-Sth-6-4	accgagcatcGAccgtataagtca	aaactgacttatacggTCgatgct
gRNA-Sth-6-5	accgagcatcagTTgtataagtca	aaactgacttatacAActgatgct
gRNA-Sth-6-6	accgagcatcagccACataagtca	aaactgacttatGTggctgatgct
gRNA-Sth-6-7	accgagcatcagccgtGCaagtca	aaactgacttGCacggctgatgct
gRNA-Sth-6-8	accgagcatcagccgtatGGgtca	aaactgacCCatacggctgatgct
gRNA-Sth-6-9	accgagcatcagccgtataaACca	aaactgGTttatacggctgatgct
gRNA-Sth-6-10	accgagcatcagccgtataagtTG	aaacCAacttatacggctgatgct

Supplementary Table 8. Primers used for IVT.

T7sgRNA-IVT-Sth1A-For	TAATACGACTCACTATAGGccatcttgtcgtctccggaaGTTTTTG
sgRNA-IVT-Sth1A-Rev	aaaaaaaAAAACACCCTGCCATAAAATGACA
T7sgRNA-IVT-Sgo-For	TAATACGACTCACTATAGGggaactcgatcgcatcattgcatgGTTTTTG
sgRNA-IVT-Sgo-Rev	aaaaaaaAAAACACCCCGCCATAAATGACAG
